# Eco-Friendly and Easily Synthesized Amorphous Fe–Ca (Oxy)hydroxide for Selective Phosphate Removal from Synthetic and Real Effluents: Synthesis, Optimization, and Application

**DOI:** 10.1021/acsomega.5c09613

**Published:** 2026-01-26

**Authors:** Yago Neco Teixeira, Elias Matias Bentes, Jackson Evangelista, Daniel Bernardes Silva, Jorge Marcell Coelho Menezes, Thiago Mielle Brito Ferreira Oliveira, Raimundo Nonato Pereira Teixeira, Ronaldo Ferreira Do Nascimento, Francisco José de Paula Filho

**Affiliations:** † Biological Chemistry Department, 226206 Regional University of Cariri, R. Cel. Antonio Luis, 1161, Crato, Ceará 63105-000, Brazil; ‡ Materials Engineering Section (SE/8), 28098 Military Institute of Engineering, R. Praça Gen. Tibúrcio, 80, Rio de Janeiro, Rio de Janeiro 22290-270, Brazil; § Science and Technology Center, 423875 Federal University of Cariri, Av. Ten. Raimundo Rocha, 1639, Juazeiro Do Norte, Ceará 63048-080, Brazil; ∥ Analytical and Physical Chemistry Department, Federal University of Ceará, Fortaleza, Ceará 60356-000, Brazil

## Abstract

The contamination of surface and groundwater by nutrients due to the discharge of untreated effluents is a serious environmental issue, as it can promote eutrophication and compromise water quality. This study aimed to optimize the synthesis of Fe–Ca (oxy)­hydroxide (CaFe) and evaluate its adsorptive capacity for phosphate ions. The coprecipitation route was chosen for the material synthesis. Structural characterizations revealed that the material exhibits high roughness, a large number of active sites, thermal stability, and a predominance of the amorphous phase, all of which favor its efficiency in phosphate adsorption. Optimization using response surface methodology (RSM) yielded a high adjusted coefficient of determination (*R*_adj_^2^ = 0.97), indicating excellent fit to the experimental data. The adsorption process followed the pseudo-second-order kinetic model (*R*_adj_^2^ > 0.99) and was best described by the Sips isotherm (*R*_adj_^2^ > 0.99), suggesting multilayer adsorption in a heterogeneous system. The maximum adsorption capacity (*q*_m_ = 41 mg/g) was achieved when the process temperature was adjusted to 30 °C. Thermodynamic parameters confirmed that the adsorption is spontaneous and endothermic (Δ*H*° = 17.38 kJ/mol). CaFe exhibits a high capacity for phosphate adsorption, even in the presence of other anions, demonstrating excellent selectivity and robustness in complex aqueous matrices. Moreover, CaFe was capable of removing up to 84.86% of phosphate from a real effluent sample by simply applying a dosage of 1.5 g/L and adjusting the effluent pH (pH = 4). In conclusion, the results demonstrated that CaFe is a promising material for phosphate removal, showing high potential for application in the treatment of water bodies susceptible to eutrophication. The findings indicated that CaFe exhibits excellent performance as an adsorbent, with high adsorption capacity, strong selectivity in the presence of competing anions, and satisfactory regeneration efficiency. Additionally, the development of sustainable materials such as CaFe is essential for achieving the Sustainable Development Goals (SDGs), particularly SDG 6, SDG 9, SDG 12, and SDG 14.

## Graphical abstract

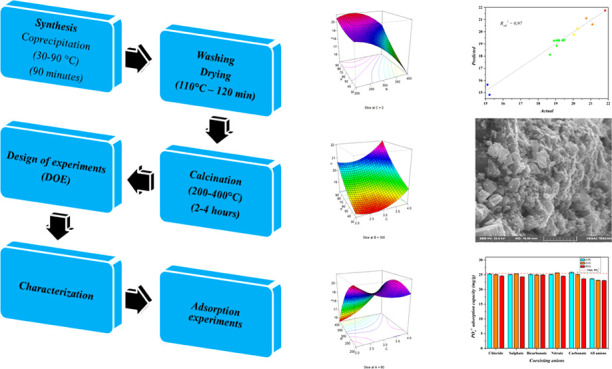

## 1 Introduction

Globally, ongoing research efforts aim to develop more streamlined and environmentally friendly synthesis routes to obtain “green materials” for various applications.^1–5^ One notable example is the use of fibers or particles derived from plant biomass as substitutes for synthetic resins in the adsorption of metals, dyes, and nutrients found in effluents and natural waters.^6–8^

In this context, technological innovations that help mitigate the harmful effects of contaminants on water resource quality represent an essential response to the current scenario of water scarcity.^9^

The discharge of untreated effluents into the environment from domestic, industrial, or agricultural sources is one of the main factors responsible for the contamination of surface and groundwater due to excessive levels of nutrients such as phosphorus (P) and its derivatives.^10^

High concentrations of phosphate ions in water can disrupt ecological balance and deteriorate water quality.^11^ The excess of this nutrient in aquatic environments poses challenges such as the planning of anthropogenic activities associated with eutrophication mitigation strategies.^12^ Nutrient-rich waters represent a valuable source of renewable resources, ready for technological exploitation. There are potential environmental benefits, including the reduction of dependence on phosphate rock and the impurities associated with its extraction and use.^13^

To date, various methods have been studied for the removal of phosphate from liquid matrices, which can be classified into three categories: chemical methods (precipitation and adsorption),^14,15^ physical methods (microfiltration and reverse osmosis),^16,17^ and biological methods (assimilation).^18^

Biological methods require strict control of pH and temperature, whereas techniques such as microfiltration and reverse osmosis involve high costs. In turn, the precipitation process may lead to the generation of large volumes of sludge, a waste that can be toxic depending on the reagents used.

Among these methods, adsorption stands out due to several advantages over other techniques, such as low operational cost, ease of handling, and high contaminant removal efficiency. Furthermore, it enables the generation of higher value-added products, such as fertilizers and agrochemicals, thereby promoting circular economy practices and environmental sustainability.

Eco-friendly, cost-effective, and easily engineered adsorbents for water treatment have gained significant popularity over the past decades. Among these are lanthanum-doped adsorbents,^19–24^ magnetic ferrites,^25–27^ modified biochars,^12,13^ and plant-based ashes.^11^

Iron- and calcium-based adsorbent materials have emerged as promising alternatives for the efficient removal of phosphate, with particular emphasis on calcium iron (oxy)­hydroxide (CaFe). This material exhibits a high chemical affinity for phosphate anions, promoting the formation of stable complexes that enhance their retention on the adsorbent surface. In addition to its adsorption efficiency, CaFe is composed of elements with low toxicity and high biocompatibility, making it environmentally safe and suitable for large-scale applications.^28,29^ Properties such as high specific surface area, the presence of active functional groups, and ion exchange capacity contribute to the material’s favorable performance. Moreover, CaFe can be synthesized through simple and low-cost methods, facilitating its scalability and application. Its ability to be regenerated and reused over multiple adsorption cycles further reinforces its potential as an efficient and sustainable solution for phosphate removal in aquatic environments.

Despite the advantages highlighted, previous studies involving iron- and calcium-based adsorbents often face significant limitations. In some formulations, although the initial adsorption capacity is high, the efficiency of phosphate recovery remains limited, with desorption rates ranging from only 15% to 39%, thereby compromising the material’s regenerability.^30^ Moreover, systems containing carbonate or bicarbonate ions can substantially reduce phosphate adsorption due to competition for active sites, particularly under alkaline conditions.^31,32^ Other studies emphasize that precipitated compounds—such as calcium carbonates formed on the adsorbent surface—may hinder both adsorption and reusability, requiring pretreatment or acid-washing steps that increase operational complexity and cost.^33^ These challenges underscore the need to develop novel Ca–Fe composites with enhanced selectivity, stability, and applicability under realistic environmental conditions.

In this context, this study aims to synthesize and evaluate a calcium–iron (oxy)­hydroxide composite for the efficient removal of phosphate from water. The novelty of this work lies in the synergistic combination of calcium and iron, which enhances not only the adsorption affinity but also the stability, selectivity, and reusability of the material. In addition to demonstrating high performance through a simple and low-cost synthesis route, the study provides new mechanistic insights into phosphate–adsorbent interactions. These findings advance the scientific understanding of adsorption processes and support the development of sustainable solutions for eutrophication control.

## 2 Materials and Methods

### 2.1 Synthesis of CaFe

The CaFe compound was synthesized using the coprecipitation method. Initially, 250 mL of NaOH solution (0.5 M) was added to a 600 mL beaker, maintained under constant stirring and temperature control using a magnetic stirrer. Then, 125 mL of FeCl_3_ solution (0.3 M) was transferred to a separatory funnel, as well as 125 mL of CaCl_2_ solution (0.19 M) to another separatory funnel. Both solutions were slowly added, dropwise, to the NaOH solution in the beaker. A schematic diagram of this system is shown in Figure 1. After the complete addition of the solutions, stirring and temperature were maintained for an additional 90 min.

**Figure 1:**
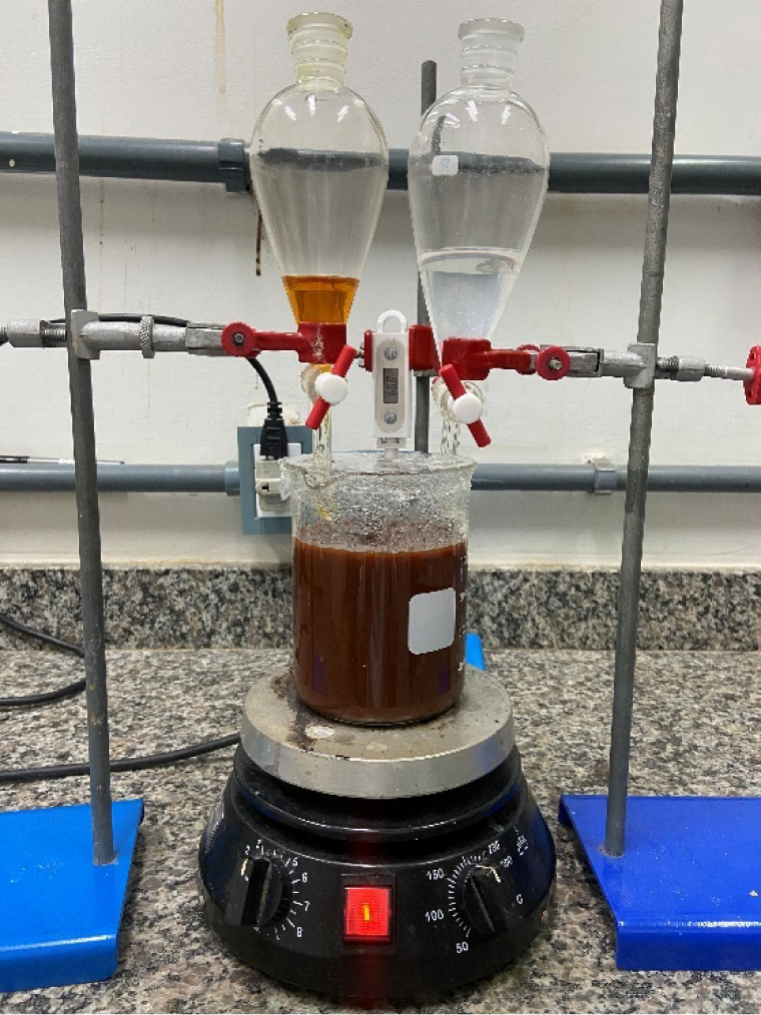
System for the synthesis of CaFe.

Subsequently, the precipitate was filtered and washed with deionized water until reaching a pH of 7. The material was then dried in an oven at 110 °C for 2 h and subsequently subjected to calcination in a muffle furnace, with temperatures ranging from 200 to 400 °C and calcination times between 2 and 4 h.

### 2.2 Design of Experiments (DOE)

The data were subjected to a response surface experiment to model the curvature of the experimental results and identify the factor settings that optimize the responses. The Box–Behnken design was selected due to its distinct advantages: it requires a smaller number of experimental runs, avoids placing experimental points at the extreme levels of all factors, and provides efficient estimation of both first- and second-order coefficients.^34^

Three different factors were used (precipitation temperature, calcination temperature, and calcination time). Each factor consisted of three levels: low (−1), high (+1), and central (0) for phosphate adsorption. A total of 17 experiments were conducted, including 12 trials and 5 central points, in order to fit the polynomial model (eq 1).

1 $$Y=\beta_{0}+\overset{n}{\underset{i=1}{\sum }}\beta_{i}x_{i}+\overset{n}{\underset{i=1}{\sum }}\beta_{ii}x_{i}^{2}+\overset{n-1}{\underset{i=1}{\sum }}\overset{n}{\underset{j=i+1}{\sum }}\beta_{ij}x_{i}x_{j}$$

Where *x*_*i*_ and *x*_*j*_ are the independent variables (precipitation temperature, calcination temperature, and calcination time), *Y* is the dependent response variable (phosphate adsorption capacity), β_*i*_, β_*ii*_, and β_*ij*_ are the regression coefficients, and β_0_ is the intercept coefficient. All results were analyzed using ANOVA with R software (version 4.2.2), RStudio (version 2023.12.1), and Minitab (version 18).

The selection of the factors evaluated in this study was based on their direct influence on the structural and surface properties of iron- and calcium-based (oxy)­hydroxide materials, such as crystallinity, surface area, and the presence of functional groups, which significantly affect phosphate adsorption capacity.

The precipitation temperature was selected based on its influence on nucleation and crystal growth processes, which affect particle size, surface area, and the distribution between amorphous and crystalline phases.^35,36^ The selected range spans from room temperature to values characteristic of mild hydrothermal synthesis, allowing for the investigation of different thermal energy regimes during the precipitation stage.

The calcination temperature was chosen due to its importance in promoting structural transformations, such as increased crystallinity, the removal of structural water, and the formation of more stable and active phases.^37^ The selected range encompasses the critical interval in which partial dehydroxylation of the hydroxides occurs without causing excessive sintering.

Calcination time was included as a factor due to its influence on phase evolution and the completeness of thermal transformations.^38^ The selected interval represents moderate durations, sufficient for the development of the desired phases without causing an excessive increase in energy consumption.

### 2.3 CaFe Characterization

The surface microstructure, morphology, and composition of CaFe were analyzed using SEM-EDS. Surface functional groups were identified by FTIR and Raman. The specific surface area (*S*_BET_) was determined using the Brunauer–Emmett–Teller (BET) equation, considering a relative pressure range of *P*/*P*_0_ = 0.02–0.30. The total pore volume (*V*_T_) was obtained at *P*/*P*_0_ = 0.98. The average pore diameter (*d*_P_) was calculated using the ratio 4000*V*_T_/*S*_BET_.

Material stability, as well as mass or component loss due to temperature variation, was assessed through TG-DTG analysis. Structural analysis was performed by XRD over a 2θ range of 20–70° using Kα radiation (λ = 1.54178 Å). The crystallite size was calculated from the peak broadening using the Scherrer equation (eq 2). The magnetic properties of the CaFe were examined by a vibrating sample magnetometer (VSM). The pH_PZC_ technique was employed to determine the point of zero charge (PZC) of CaFe. For this purpose, the solid addition method was used.^2^ Briefly, six KCl solutions (0.1 M) with pH values ranging from 2 to 12 and a volume of 50 mL each were prepared. A mass of 0.01 g of CaFe was added to each sample, and the mixtures were stirred for 24 h (150 rpm, 30 °C). HCl (1 M) and NaOH (1 M) solutions were used to adjust the pH.

2 $$D=\frac{0.9\lambda }{\beta \cos\theta }$$

In eq 2, *D* represents the average crystallite size perpendicular to the reflecting planes, λ represents the X-ray wavelength, β represents the full width at half-maximum (fwhm), and θ is the diffraction angle.

### 2.4 Adsorption Experiments

Table 1 provides all the values and parameters analyzed in the batch adsorption tests.

**Table 1:** Adsorption Experiments Analyzed

Test	CaFe dosage (g/L)	Phosphate concentration (mg/L)	Coexisting anions (mg/L)	Time (min)	pH	Temperature (°C)	Volume (mL)
Adsorbent dosage	0.5, 1.5, 2.5, 3.5, 5, 6	10, 50, 100	0	120	4	30	25
Isotherms and Termodynamics Adsorption	1.5	10, 30, 50, 80, 100	0	120	4	30, 40, 50	25
Kinetics Adsorption	1, 1.5, 5	10, 50, 100	0	0, 3, 5, 10, 20, 30, 60, 90, 120	4	30	50
Coexisting anions	1.5	50	10, 50, 250.	120	4	30	25

A stirring speed of 150 rpm was used for all experiments. CaFe was separated from the solution with the aid of an external magnetic field after the completion of each experiment. The residual phosphate concentration in the samples was analyzed using the Murphy and Riley method,^39^ employing a UV–vis spectrophotometer (Shimadzu, UV 1800) with a fixed wavelength of 882 nm. In summary, this procedure—also known as the phosphomolybdate method—is based on the reaction of the phosphate ion with ammonium molybdate in an acidic medium, forming phosphomolybdic acid. In the following step, ascorbic acid reduces this compound, generating a blue-colored complex whose intensity is directly proportional to the phosphate concentration. The adsorption capacities were calculated using eqs 3 and 4. The removal efficiency was calculated using eq 5

3 $$q_{t}=\left(\frac{C_{o}-C_{t}}{m}\right)V$$

4 $$q_{e}=\left(\frac{C_{o}-C_{e}}{m}\right)V$$

5 $$\mathrm{Removal}=\left(\frac{C_{o}-C_{e}}{C_{o}}\right)100$$

Where: *q*_t_ is the adsorption capacity at a given time (mg/g), *q*_e_ is the adsorption capacity at equilibrium time (mg/g), Removal is the removal efficiency of phosphate (%), *C*_o_ is the initial concentration of phosphate (mg/L), *C*_t_ is the concentration of phosphate at a given time (mg/L), *C*_e_ is the concentration of phosphate at equilibrium time (mg/L), *m* is the mass of CaFe used (g), and *V* is the volume of the solution (L).

### 2.5 Kinetic, Isotherm, and Thermodynamic Modeling of Adsorption

The analysis of kinetic models is essential to evaluate the mass transfer over a specified contact period, as well as to understand the adsorption mechanisms involved in the process. For this study, the pseudo-first-order (PFO), pseudo-second-order (PSO), and Elovich kinetic models were used. The equations for the PFO, PSO, and Elovich models are described by eqs 6, 7 and 8, respectively.

6 $$q_{t}=q_{e}\left(1-e^{-k_{1}t}\right)$$

7 $$q_{t}=\frac{{q_{e}}^{2}k_{2}t}{1+q_{e}k_{2}t}$$

8 $$q_{t}=\frac{1}{\beta }\ln\left(1+\alpha \beta t\right)$$

Where: *k*_1_ and *k*_2_ are the adsorption rate constants for PFO (min^–1^) and PSO (g·mg^–1^·min^–1^), respectively, α is the initial adsorption rate (mg·g^–1^·min^–1^), β is the desorption constant (mg/g), and *t* is the time (min).

The study of adsorption isotherm models is essential to quantify the maximum adsorption capacity of an adsorbent, as well as to indicate the type of adsorption occurring (physisorption or chemisorption), the optimal temperature, and the feasibility of the process. For this study, the Langmuir Isotherm model (eq 9), Freundlich model (eq 10), Sips model (eq 11), and Dubinin–Radushkevich (DR) model (eq 12) were used.

9 $$q_{e}=\frac{q_{m}k_{L}C_{e}}{1+k_{L}C_{e}}$$

10 $$q_{e}=k_{F}{C_{e}}^{1/n}$$

11 $$q_{e}=\frac{q_{m}k_{S}{C_{e}}^{n}}{1+k_{S}{C_{e}}^{n}}$$

12 $$q_{e}=q_{m}\exp\left\{-k_{DR}{[RT\ln\left(1+\frac{1}{C_{e}}\right)]}^{2}\right\}$$

Where: *k*_L_, *k*_F_, *k*_S_, and *k*_DR_ are the isotherm constants for the Langmuir (L/mg), Freundlich (mg·g^–1^ ·(mg·L^–1^)^−1^/^*n*^), Sips (L/mg), and Dubinin–Radushkevich (mol^2^/kJ^2^) models, respectively. *n* is the constant related to the heterogeneity of the adsorbent surface. *q*_m_ is the maximum monolayer adsorption capacity (mg/g). *R* is the universal gas constant (8.314 J·mol^–1^ ·K^–1^). *T* is the process temperature (K).

The constant *k*_DR_ is associated with the mean adsorption energy, which can be calculated using eq 13

13 $$E=\frac{1}{\sqrt{2k_{\mathrm{DR}}}}$$

Where: *E* is the mean adsorption energy of the process (kJ/mol). Based on the value of *E*, the nature of the process can be assessed: physisorption (*E* < 8 kJ/mol), ion exchange (8 < *E* < 16 kJ/mol), or chemisorption (*E* > 16 kJ/mol).^40^

The study of adsorption thermodynamics is essential to identify the nature of the process (endothermic or exothermic), as well as its spontaneity. The thermodynamic parameters were obtained from the Van’t Hoff equation (eq 14) and the Gibbs free energy change equation (eq 15).

14 $$k_{S}=\exp\left[\frac{\Delta S^{{}^{\circ}}}{R}-\left(\frac{\Delta H^{{}^{\circ}}}{R}\right)\frac{1}{T}\right]$$

15 $$\Delta G^{{}^{\circ}}=\Delta H^{{}^{\circ}}-T\Delta S^{{}^{\circ}}$$

Where: Δ*H*° is the enthalpy change (kJ/mol), Δ*S*° is the entropy change (kJ/mol), Δ*G*° is the Gibbs free energy change (kJ/mol), and *k*_S_ is the equilibrium constant from the Sips isotherm (dimensionless). To render the equilibrium constant dimensionless, it was assumed that the activity coefficient of the dilute solution is equal to one. The nonlinear equations of each model were used to calculate all parameters.

### 2.6 Desorption Tests

Desorption tests are necessary when aiming to identify the most effective eluent for promoting desorption between the adsorbate and the adsorbent. Additionally, this analysis serves as a basis for regeneration studies (adsorption–desorption cycles). The following eluents were evaluated: NaOH (0.1 M), NaOH (0.5 M), NaCl (0.1 M), NaCl (0.5 M), and deionized water. The desorption efficiency of the eluents was calculated using eq 16

16 $$\mathrm{Desorption}=\left(\frac{q_{\mathrm{des}}}{q_{e}}\right)100$$

Where *q*_des_ is the amount of phosphate desorbed (mg/g) after the contact between CaFe and the eluents. For this test, the following conditions were used in the adsorption step prior to desorption: CaFe dosage of 1.5 g/L, initial phosphate concentration of 50 mg/L, pH of 4, agitation at 150 rpm, and temperature of 30 °C.

### 2.7 Application in Real Effluent

CaFe was used to remove phosphate from effluent samples collected from the municipal slaughterhouse of Juazeiro do Norte, Brazil (7°13′12.8″S 39°20′48.7″W). Prior to the adsorption process, the samples underwent preliminary treatment followed by biological treatment in a UASB reactor. The following effluent parameters were analyzed before and after adsorption: pH, conductivity, turbidity, temperature, and phosphate concentration.

## 3 Results and Discussion

### 3.1 Material Characterizations

The morphology and composition of CaFe before and after phosphate adsorption were analyzed using SEM-EDS. As shown in Figure 2a, CaFe exhibits a broad particle size distribution, ranging from 500 μm to less than 5 μm. Its morphology is notably complex, with numerous smaller particles of irregular geometric shapes agglomerated on the surface of larger particles, resulting in a rough surface texture (Figure 2b,c). This agglomeration can be attributed to self-magnetic forces among CaFe particles, leading to the formation of large aggregates. Furthermore, the presence of defects and voids on the material surface suggests a high surface area and pore volume features that are highly desirable in an adsorbent material. Similar results were reported by Xia et al. in their study on the removal of phosphate from aqueous solutions using manganese ferrites.^27^

**Figure 2:**
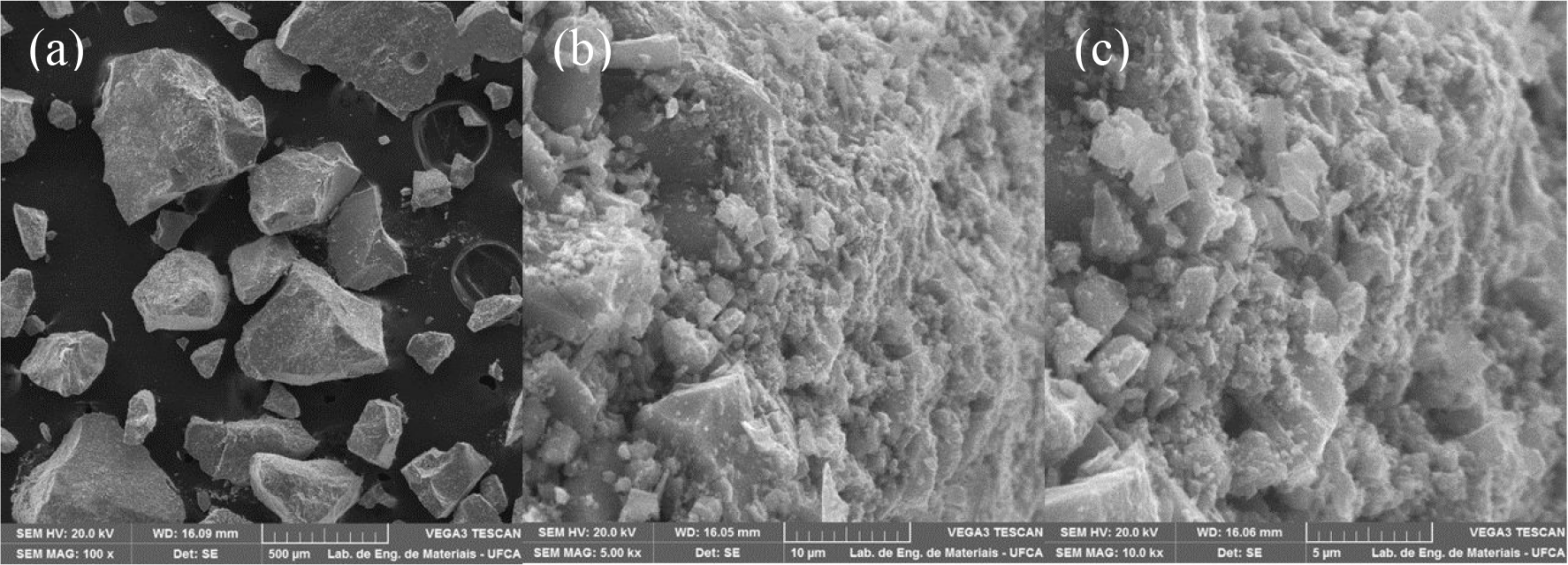
Morphological analysis of the CaFe produced, after amplification of (a) 100×, (b) 5000× and (c) 10000×.

The Figure 2c reveals the presence of nanoscale crystallites distributed over the surface, forming a heterogeneous layer with significant roughness and porosity. Such features increase the number of accessible active sites, thereby enhancing surface reactivity and facilitating both surface complexation and precipitation mechanisms of phosphate binding. The coexistence of micro- and nanoscale structures is particularly advantageous because larger particles provide structural stability, while the finer particles increase the effective surface area and adsorption capacity. In this context, the observed morphology of CaFe represents an innovative advantage, as it not only improves phosphate capture but also contributes to material stability and reusability in cyclic adsorption processes.

The chemical composition and elemental mapping of CaFe before and after phosphate adsorption are illustrated in Figure 3. The dominant elements are Fe, O, and Ca, with Fe being the major constituent (≈73%). Following the adsorption experiments, the element P appears in the composition and is distributed across the particle surfaces in the elemental mapping, confirming the ability of CaFe to adsorb phosphate ions.

**Figure 3:**
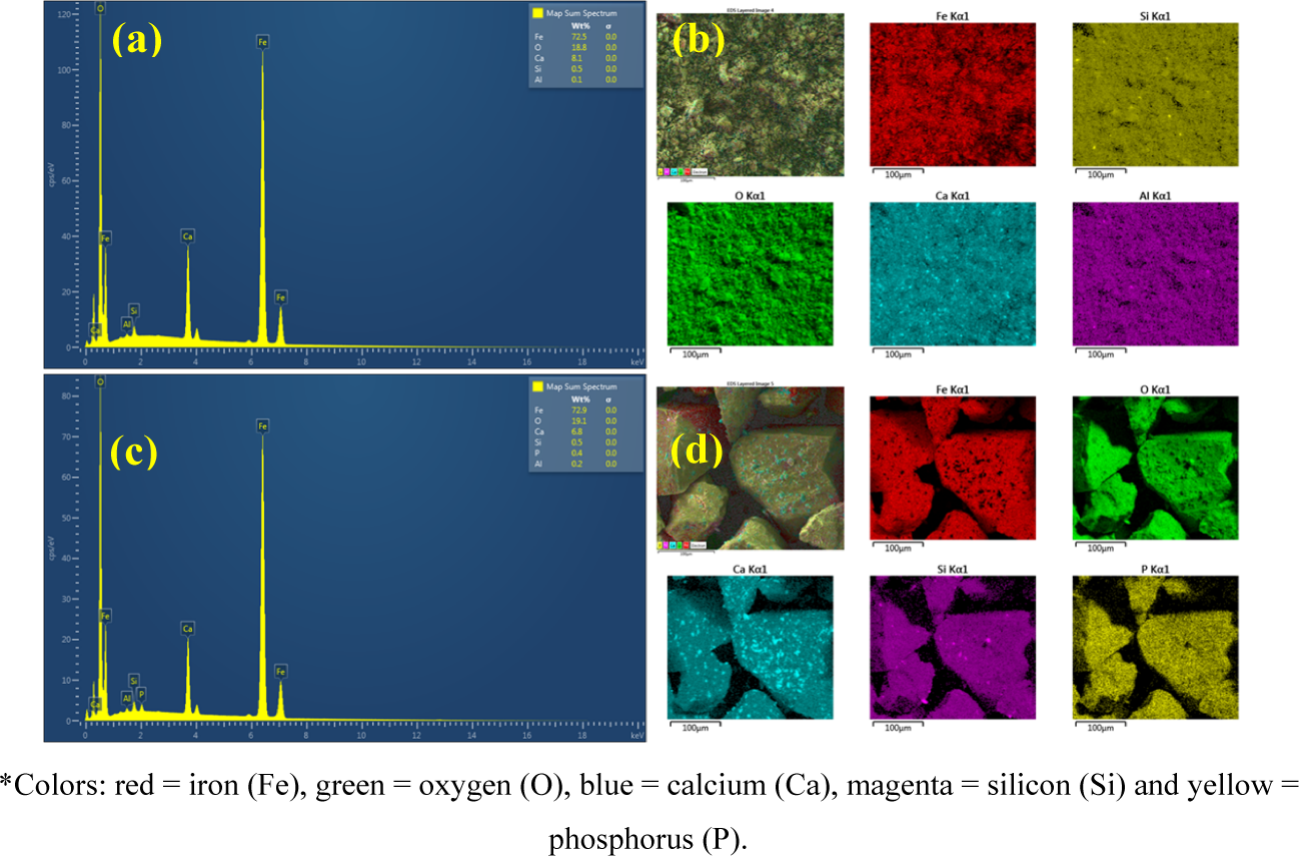
EDS results. (a) Elemental composition of CaFe before the phosphate adsorption process; (b) Elemental mapping of CaFe before the phosphate adsorption process; (c) Elemental composition of CaFe after the phosphate adsorption process; (d) Elemental mapping of CaFe after the phosphate adsorption process.

Through FTIR analysis (Figure 4), the main functional groups present on the surface of CaFe were identified. The weak absorption bands detected between 2922 and 2852 cm^–1^ correspond to CH stretching, likely from residual organic groups or atmospheric CO_2_. Peaks in the region 1795–1746 cm^–1^ and at 1616 cm^–1^ are associated with CO_3_^2–^ vibrations and bending of water molecules, respectively.^41^ The carbonate-related bands are particularly relevant because CO_3_^2–^ species often compete with phosphate for adsorption sites on Ca-based materials. The peaks at 1411–1378 cm^–1^ are associated with the incorporation of CO_3_^2–^, which originates from the synthesis process. This process involves a high concentration of OH^–^ ions, which exhibit strong affinity for CO_3_^2–^, particularly under alkaline conditions. Consequently, this suggests that part of the HPO_4_^2–^ may be replaced by CO_3_^2–^.

**Figure 4:**
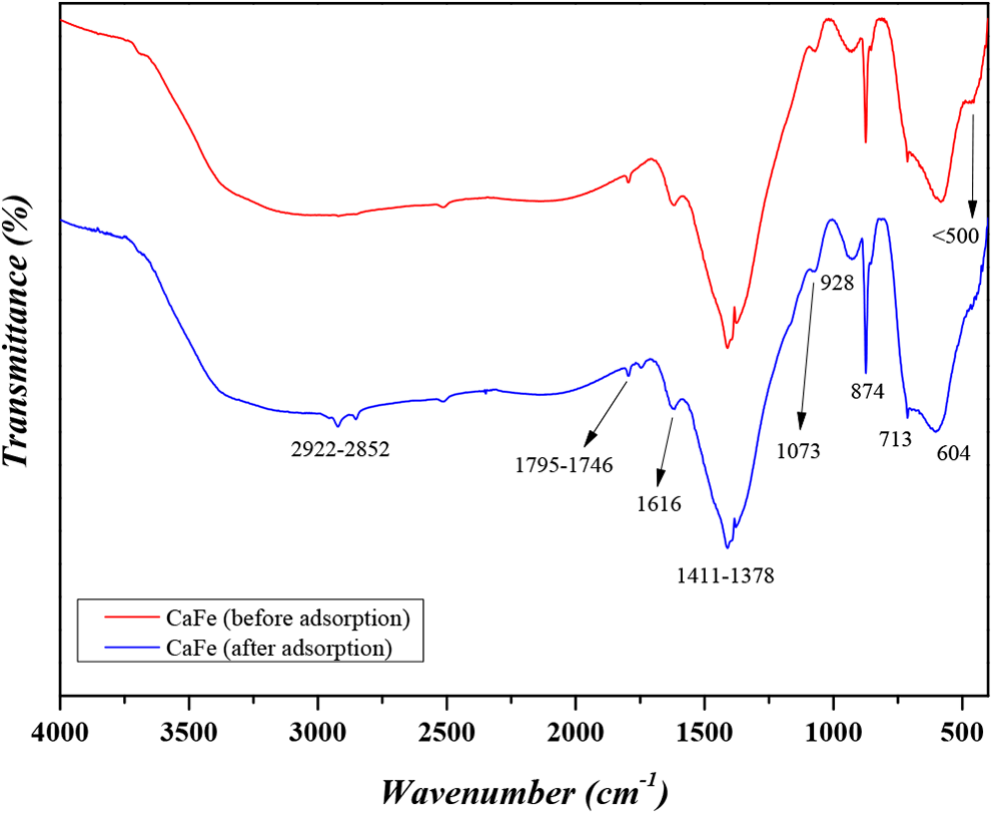
Surface functional groups of CaFe before and after the phosphate adsorption process.

Previous studies have also demonstrated that CO_3_^2–^ can compete with phosphate for active adsorption sites on Fe–Ca composites, thereby reducing phosphate uptake efficiency and potentially affecting the stability of the adsorbed species.^30–33^ These findings highlight the importance of considering carbonate interference when designing Ca–Fe-based adsorbents for realistic environmental conditions.

Notably, the intensification of absorption bands at 1073, 928, and 874 cm^–1^ in the postadsorption spectrum are characteristic of P–O stretching vibrations of phosphate groups. The band at 1073 cm^–1^ corresponds to the symmetric stretching of PO_4_^3–^ , but it has also been associated with Fe–Ca stretching, suggesting that this signal arises both from PO_4_^3–^ binding and intrinsic structural vibrations of the composite.

Similarly, the peak at 928 cm^–1^ can be attributed to HPO_4_^2–^ species or secondary phosphate interactions on the adsorbent surface, while also being related to CaOH vibrations. The band at 874 cm^–1^ is assigned to HPO_4_^2–^ and secondary P–O modes, but it also reflects FeOH bending, supporting the confirmation of Fe–Ca bonds and the possible incorporation of CO_3_^2–^ on the material surface. Additional features at 713 and 604 cm^–1^ further support phosphate incorporation through P–O bending vibrations, while also being associated with Fe–O stretching modes characteristic of CaFe_2_O_4_ spinel nanoparticles.

The bands in the fingerprint region below 500 cm^–1^ are assigned to Fe–O and Ca–O lattice vibrations. The preservation of these signals before and after adsorption demonstrates the structural stability of the Fe–Ca framework, while the appearance of new phosphate-related bands indicates that the adsorption process occurs primarily via surface complexation and precipitation reactions involving Ca^2+^ and Fe^3+^ active sites.^42,43^

These findings highlight the synergistic role of Ca and Fe in phosphate removal: Ca promotes precipitation of calcium phosphates, while Fe facilitates surface complexation through FeOH groups. The combined mechanism not only enhances adsorption affinity but also contributes to the stability of the retained phosphate species. Compared to conventional Fe- or Ca-only adsorbents, the presence of both elements broadens the spectrum of available interaction pathways, which represents an innovative advantage of the Ca–Fe (oxy)­hydroxide composite developed in this study.

The thermal stability of CaFe was investigated through thermogravimetric analyses in a nitrogen atmosphere (Figure 5). The first mass loss event, around 100 °C, was attributed to the removal of water from the surface of CaFe. The second mass loss event, around 200 °C, was related to the elimination of carbon dioxide adsorbed on the surface of CaFe. The total mass loss observed in the studied temperature range was approximately 14%. Notably, above 300 °C, no new peaks were identified in the DTG curve behavior, indicating the absence of impurities. Similar results were obtained by Khanna and Verma when synthesizing and characterizing calcium ferrite for cytotoxicity analyses.^44^

**Figure 5:**
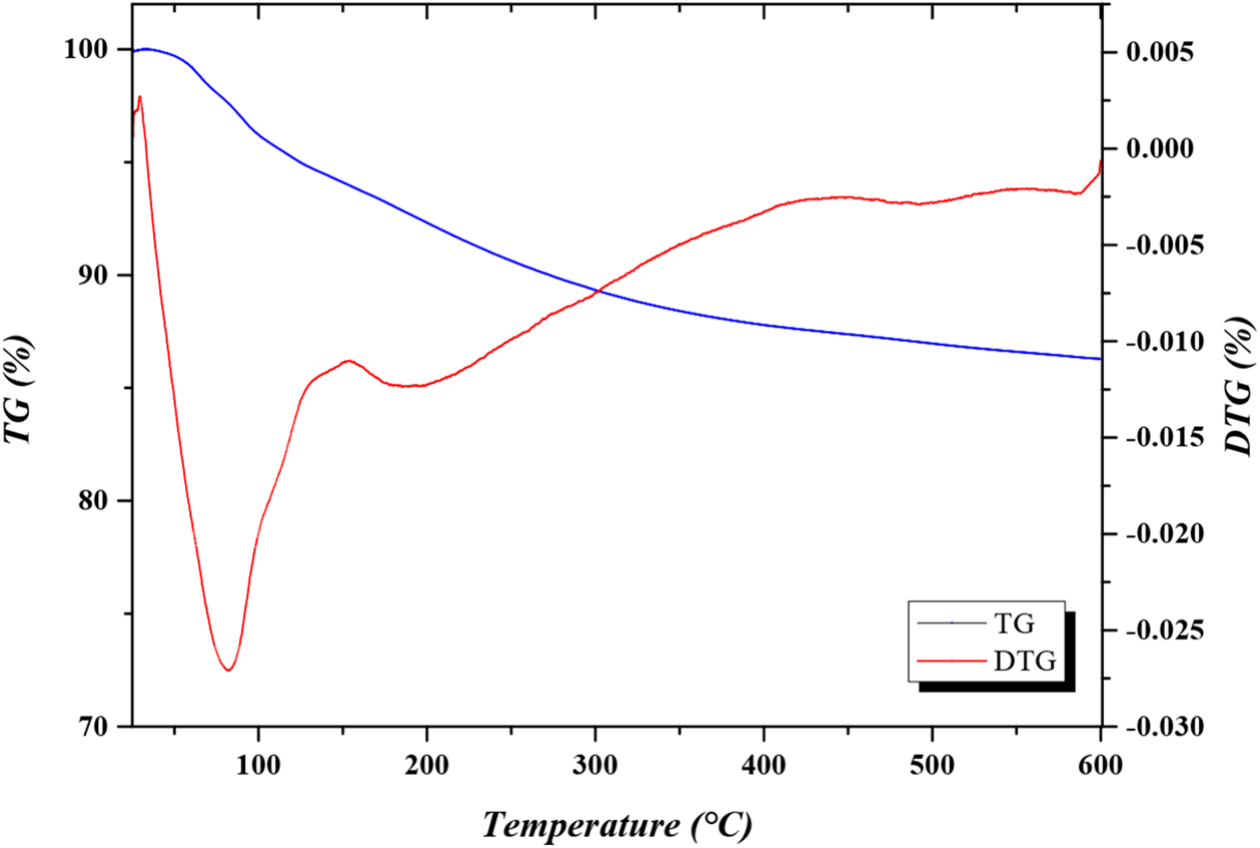
Thermal stability analysis of CaFe.

Figure 6 presents the XRD pattern for CaFe. The obtained data indicate that the material has a predominantly amorphous structure with minor crystalline domains, with the formation of three distinct phases: Ca_2_Fe_2_O_5_, Ca_2_Fe_9_O_13_, and Fe_2_O_3_, each with specific characteristics. Based on the Scherrer equation (eq 2), the average crystallite size was estimated to be 45.74 nm.

**Figure 6:**
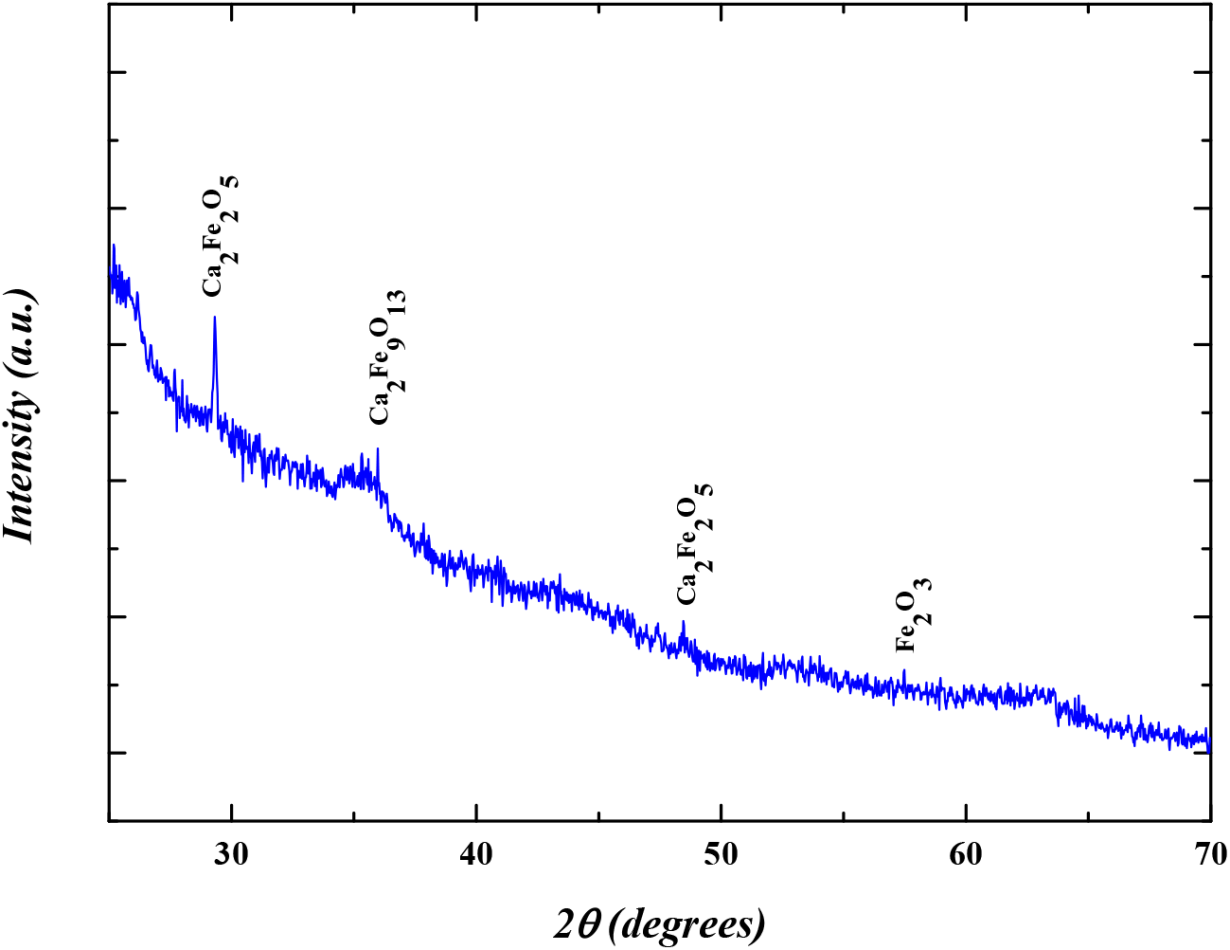
XRD analysis results.

The presence of Ca_2_Fe_2_O_5_ and Ca_2_Fe_9_O_13_ suggests that the material exhibits a mixed Ca–Fe oxide framework, which is known to enhance surface reactivity due to the coexistence of multiple cationic species. These mixed oxides provide abundant active sites and contribute to synergistic effects, where Ca^2+^ favors precipitation of phosphate species, while Fe^3+^ facilitates inner-sphere complexation via Fe–O–P bonding.^45,46^

Amorphous regions are particularly beneficial in adsorption processes because they offer higher structural flexibility and more unsaturated coordination sites compared to highly crystalline phases, thus improving adsorption capacity. Therefore, the XRD results highlight that the synergistic contribution of crystalline Ca–Fe phases embedded in an amorphous matrix provides a promising structural feature for efficient and stable phosphate adsorption.

Figure 7 presents the Raman spectroscopy results for CaFe. In the Raman spectrum, the bands observed at 211 and 270 cm^–1^ are attributed to vibrational modes associated with Fe–O bonds in iron oxides and iron ferrites, consistent with the E_g_ and T_2g_ modes reported for hematite and calcium ferrites. The band at 702 cm^–1^ can be related to lattice modes involving Fe–O–Fe and Fe–O–Ca linkages, which are typical of ferrite-type structures and mixed oxides, corroborating the phases identified by XRD. The intense peak observed at 1063 cm^–1^ is assigned to the symmetric stretching mode of the carbonate group, indicating the presence of surface carbonation of calcium, a phenomenon commonly observed in materials containing CaO or Ca­(OH)_2_ exposed to atmospheric CO_2_. This assignment is strongly supported by the FTIR results.^47–49^

**Figure 7:**
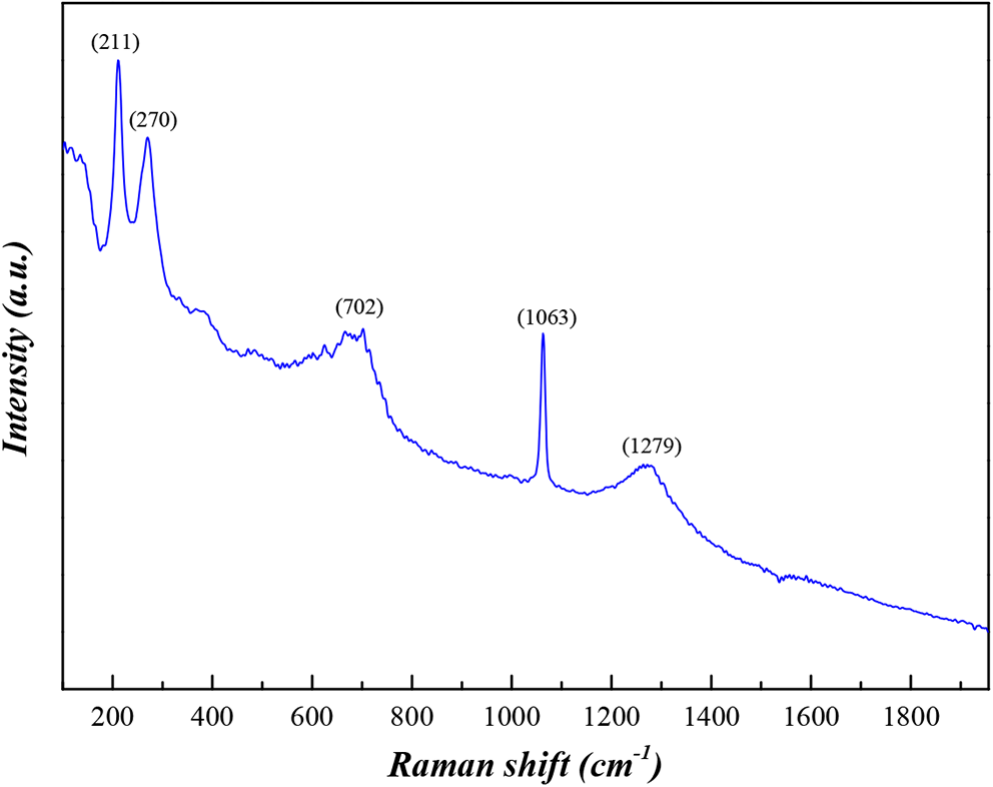
Raman spectroscopy of CaFe.

Overall, the Raman, FTIR, and XRD results demonstrate that the composite consists of a matrix rich in iron oxides and calcium ferrites, with surface carbonation of calcium, forming active sites that are favorable for subsequent interaction with phosphate in solution. This structural composition is particularly relevant for phosphate removal applications, as it combines mechanisms of surface adsorption on iron oxides with the possible precipitation and/or complexation with calcium species after contact with the effluent.

Figure 8 presents the N_2_ adsorption–desorption isotherms of the CaFe material. As observed, the isotherm curve is classified as type IV with H3 hysteresis, indicating a predominance of mesoporous behavior associated with a nonuniform pore size distribution and pore connectivity. The BET surface area (*S*_BET_) and total pore volume (*V*_T_) were 272 m^2^/g and 0.17 cm^3^/g, respectively. The desorption isotherm was used to determine the pore size distribution, with an average pore diameter (*d*_p_) of approximately 2.5 nm.

**Figure 8:**
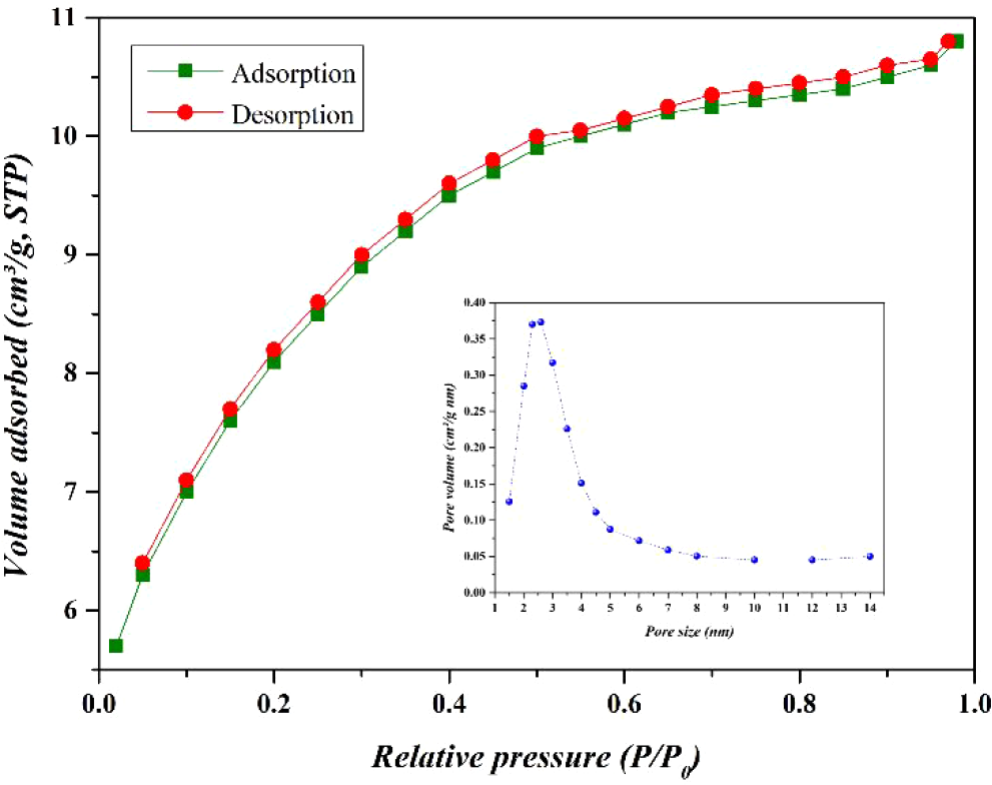
N_2_ adsorption/desorption isotherms and pore volume distribution of CaFe.

The magnetic response of the CaFe was studied by VSM measurements in the magnetic field of −14 K to +14 K Oe (Figure 9). The sample exhibits superparamagnetic behavior, with a saturation magnetization (Ms) value of 3.50 emu/g. The remanent magnetization (MR) and the squareness ratio (MR/Ms) are 0.13 emu/g and 0.071, respectively. The superparamagnetic behavior is confirmed by the MR/Ms ratio being lower than 0.1, indicating that the synthesized nanoparticles can be magnetized in the presence of an external magnetic field, enabling faster and more efficient separation from aqueous solutions.

**Figure 9:**
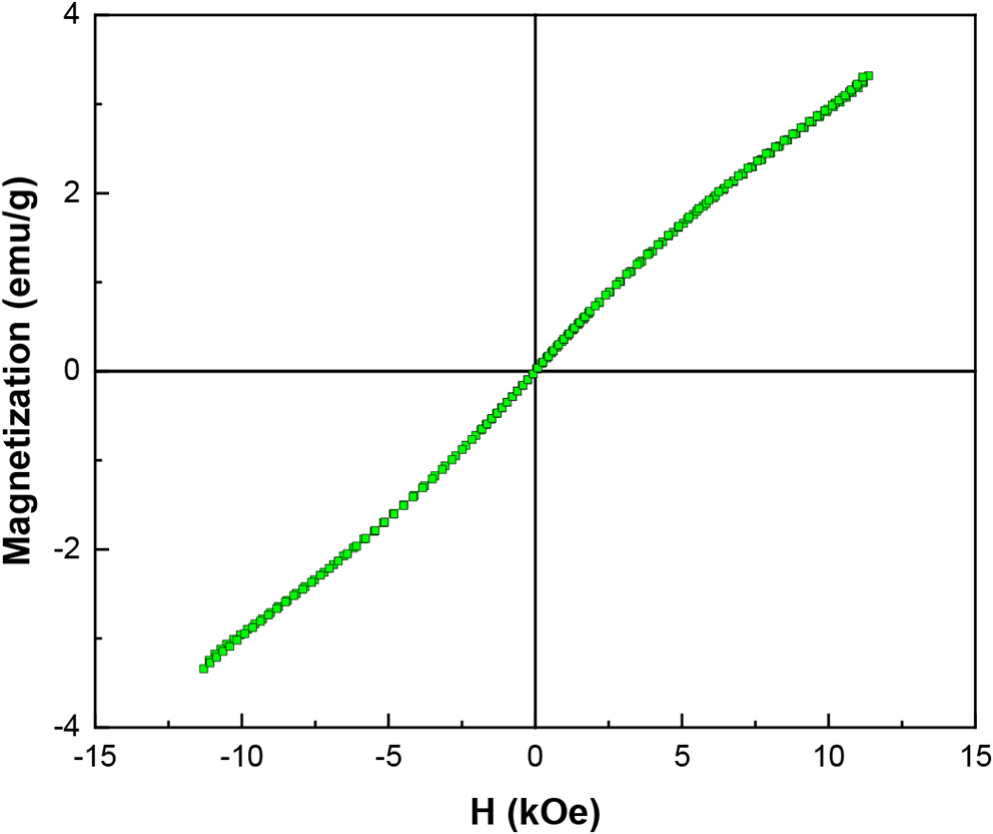
VSM magnetization curve of CaFe.

In order to determine the pH value at which CaFe has no net charge, a zero charge point (pH_PZC_) analysis was performed. From Figure 10, it can be concluded that the pH_PZC_ value of CaFe is 6.8. Therefore, to favor the interaction between CaFe and phosphate, the pH of the medium should be below 6.8, as this will cause the surface of CaFe to be positively charged, enhancing the CaFe-phosphate interaction. Consequently, all adsorption experiments were conducted at a pH of 4, where phosphate is predominantly in the form of dihydrogen phosphate (H_2_PO_4_^–^), exhibiting a strong affinity for CaFe, thereby increasing adsorption capacity and removal efficiency.

**Figure 10:**
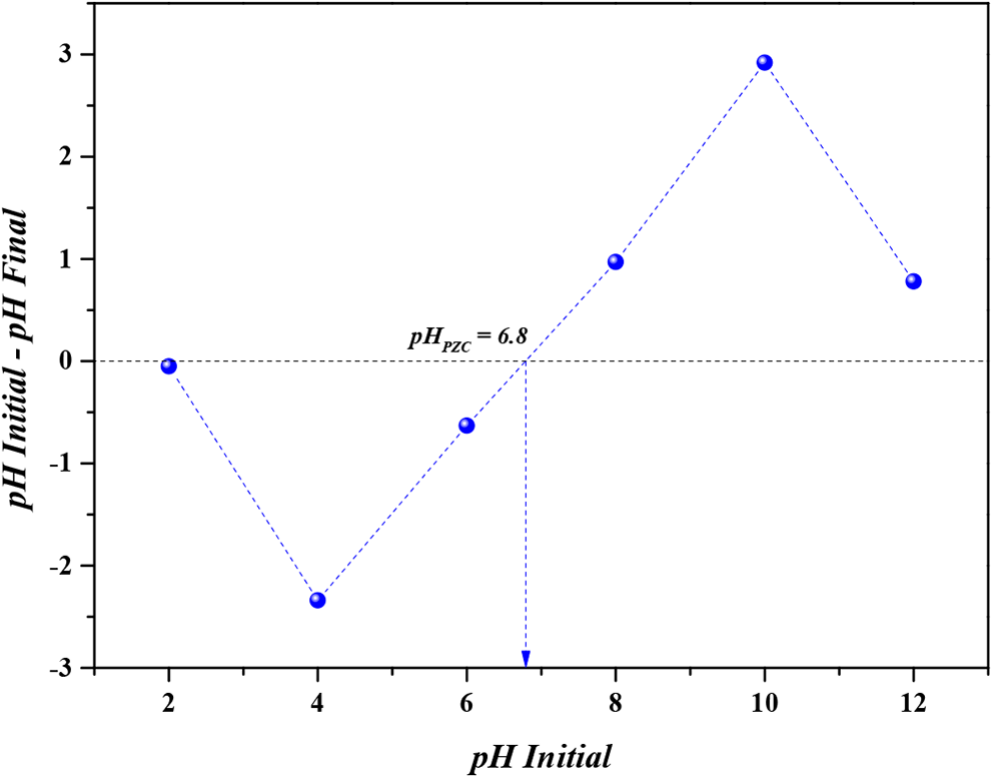
Analysis of the zero charge point of CaFe.

At pH values lower than 4, partial dissolution of CaFe may occur, while a basic medium (pH > 7) promotes competition between phosphate and OH^–^, reducing adsorption capacity and removal efficiency. Similar results were observed by Brontowiyono, Gu and colleagues when analyzing the removal of phosphate from aqueous solutions using manganese and zinc ferrites, respectively.^25,26^

### 3.2 Optimization Using RSM

The results of the adsorption capacity of CaFe under different conditions are presented in Table S1. The values obtained for adsorption capacity ranged between 15 and 22 mg/g. The synthesis conditions that resulted in the lowest adsorption capacity were as follows: precipitation temperature of 60 °C, calcination temperature of 400 °C, and calcination time of 2 h. The synthesis conditions that resulted in the highest adsorption capacity were as follows: precipitation temperature of 90 °C, calcination temperature of 300 °C, and calcination time of 4 h.

The analysis of variance (ANOVA—-Table S2), combined with a Pareto chart (Figure 11), was performed to identify the synthesis parameters that most impact the adsorption of phosphate. Based on Figure 11, it can be concluded that the parameter with the greatest impact on maximizing the adsorption capacity of phosphate is the calcination temperature (*B*), followed by the precipitation temperature (*A*). The calcination time (*C*) also plays a critical role in the process. However, the interactions between temperature and calcination time (*BC*), as well as the quadratic interaction between calcination temperature (*BB*), have a more pronounced effect on the process. The noncritical factors were the interaction between precipitation temperature and calcination time (*AC*), and the quadratic interaction between precipitation temperature (*AA*).

**Figure 11:**
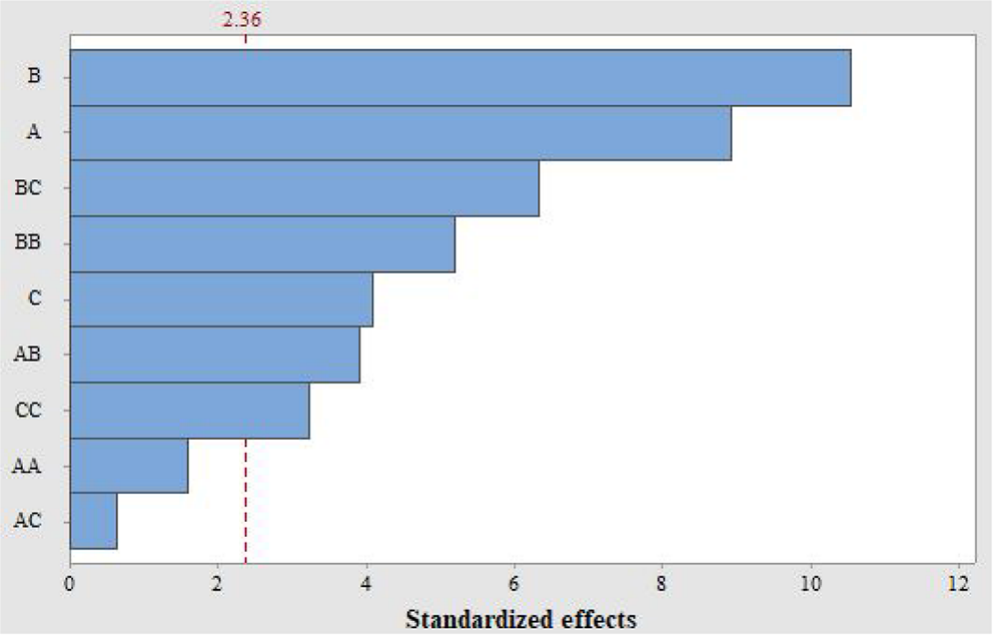
Pareto chart for analysis of significant effects.

Table S2 is consistent with the results presented in Figure 11, as the p-values of the parameters corroborate with the Pareto chart. In summary, the lower the p-value presented in Table S2, the more critical the parameter is for the process.

The critical influence of calcination temperature on PO_4_^3–^ adsorption can be explained by the variety of crystalline arrangements that CaFe can exhibit, depending on the synthesis temperature. This variation directly affects the number of available active sites and functional groups. Iron, due to its malleability at different temperatures, can rearrange the material, leading to different phases such as ferrite, hematite, goethite, magnetite, and maghemite, each with distinct adsorption properties.

In order to improve the polynomial equation modeling for the process, the noncritical factors (*A* ^2^ and *AC*) were removed. This resulted in new determination coefficient values: *R* ^2^ = 0.9677, *R*_adj_^2^ = 0.9426, and *R*_pred_^2^ = 0.7939. This action significantly improved the model’s ability to predict the results, with a 13.8% increase in *R*_pred_^2^. The equation of the model in unscaled units is presented in eq 17

17 Y=32.30−0.0357A−0.0088B−6.99C−0.000098B∗B+0.638C∗C+0.000257A∗B+0.01245B∗C

Table S3 presents the experimental values and the predicted values using the polynomial equation of the reduced model. Figure 12 illustrates the model’s ability to predict the phosphate adsorption capacity by CaFe, showing a prediction capacity within the studied range of 97%.

**Figure 12:**
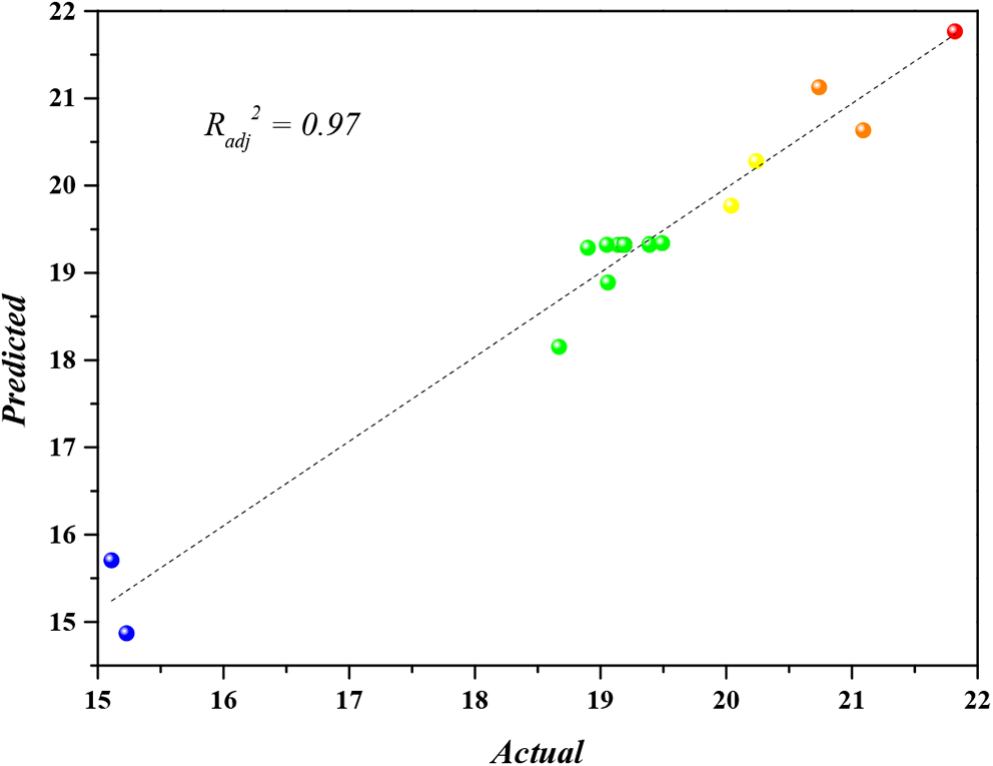
Regression of the prediction model from the experimental results.

In Figure 13, the first parameters to be evaluated were the effects of precipitation and calcination temperatures (A1). It can be concluded that increasing the system temperature during the precipitation step enhances the phosphate adsorption capacity. This can be attributed to improved crystallinity control and the generation of reactive surface hydroxyl groups, which favor phosphate complexation with Ca^2+^ and Fe^3+^ active sites. The opposite behavior is observed with respect to the calcination temperature, meaning that increasing the calcination temperature hinders phosphate adsorption. Higher temperatures promote particle sintering and reduce surface area and porosity, thereby limiting the accessibility of adsorption sites.

**Figure 13:**
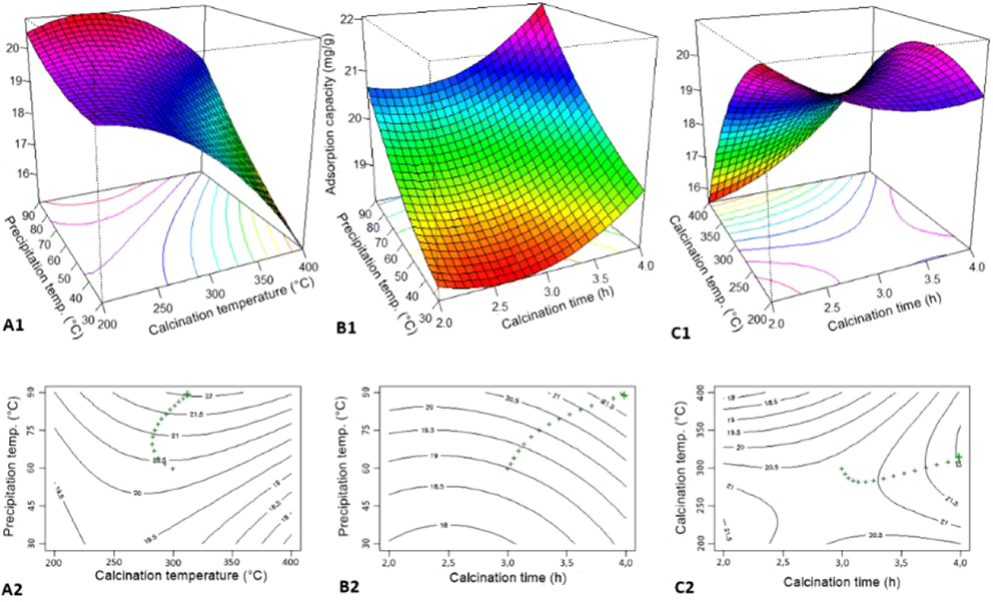
Response surfaces and restricted optimization for optimization of phosphate adsorption on CaFe.

The effects of precipitation temperature and calcination time (B1) indicate that increasing the calcination time increases the phosphate adsorption capacity. This is likely due to enhanced stabilization of Ca–Fe oxide phases and the removal of structural defects that could otherwise compromise surface reactivity.

The effects of calcination temperature and calcination time (C1) exhibit a saddle behavior, meaning there is neither a minimum nor a maximum point. Such nonlinear behavior is common in multivariable optimization processes where competing mechanisms—such as surface area reduction versus structural stabilization—are simultaneously at play.

Therefore, restricted optimization was applied, as shown in Figure 13 (A2, B2, and C2), where it was found that to optimize the synthesis, CaFe should be precipitated at 90 °C and calcined for 4 h at a temperature of 313 °C. These conditions balance crystallinity, porosity, and active site preservation, yielding a composite with high selectivity and affinity for phosphate species. This optimization highlights the importance of carefully tailoring synthesis parameters to maximize environmental performance.

### 3.3 Adsorption Kinetics and Dosage Effect

The results of the adsorption kinetics are presented in Figure 14. It is observed that increasing the CaFe dosage reduces the time required to reach equilibrium at phosphate concentrations of 50 and 100 mg/L. With a dosage of 1.5 g/L of CaFe, equilibrium was reached in 60 min, while for 5 g/L, the time reduced to 20 min at a concentration of 50 mg/L and 30 min at 100 mg/L. For the concentration of 10 mg/L of phosphate, there were no significant changes in the equilibrium time for both dosages analyzed. After equilibrium, no significant increase in the adsorption capacity was observed.

**Figure 14:**
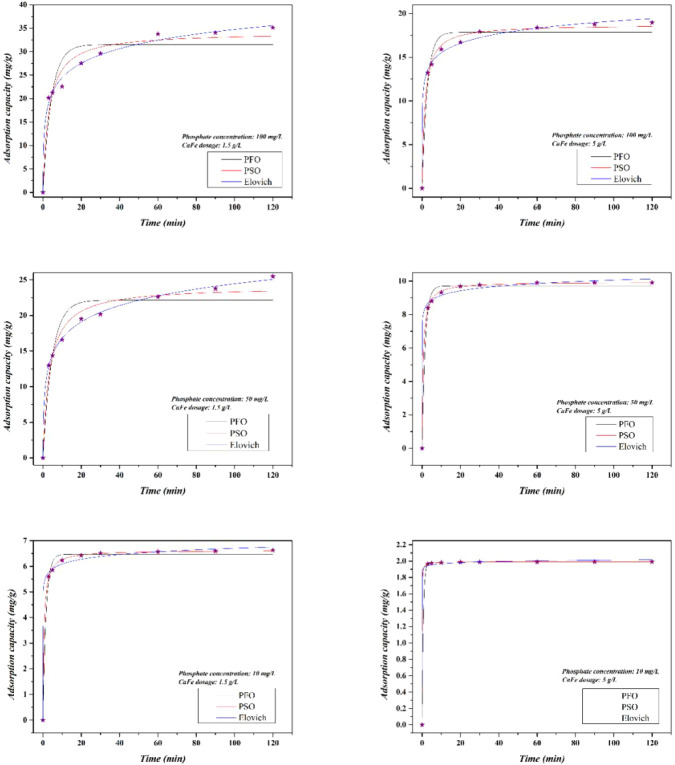
Adsorption capacity vs contact time for different dosages of CaFe and different concentrations of phosphate.

Increasing the CaFe dosage provides greater availability of active sites for adsorption, which accelerates the capture of phosphate molecules and consequently reduces the time required to reach equilibrium. This acceleration is explained by the enhanced probability of interactions between phosphate ions and adsorption sites, which decreases the mass transfer driving force more rapidly and leads to shorter equilibrium times. However, it should be emphasized that higher dosages, while beneficial for adsorption kinetics, do not always result in a proportional increase in adsorption capacity per unit mass. This limitation arises from particle aggregation and overlapping of active sites, underscoring the importance of optimizing adsorbent dosage to balance kinetics and material efficiency for practical applications.

The adsorption kinetic modeling data are presented in Table S4. The PSO and Elovich models provided the best fit to the experimental data, exhibiting the highest *R*_adj_^2^ values and the lowest RMSE values. Furthermore, the predicted *q*e values from the PSO model were very close to the experimental values, further indicating that this model provided a good fit. The good fit of the Elovich model further suggests that the adsorption process occurs on a heterogeneous surface with a wide distribution of activation energies. Together, these findings highlight that the phosphate adsorption mechanism involves both specific surface interactions and heterogeneous energetic sites.

### 3.4 Adsorption Isotherms

The behavior of the adsorption isotherms is illustrated in Figure 15, and the modeling results are presented in Table S5.

**Figure 15:**
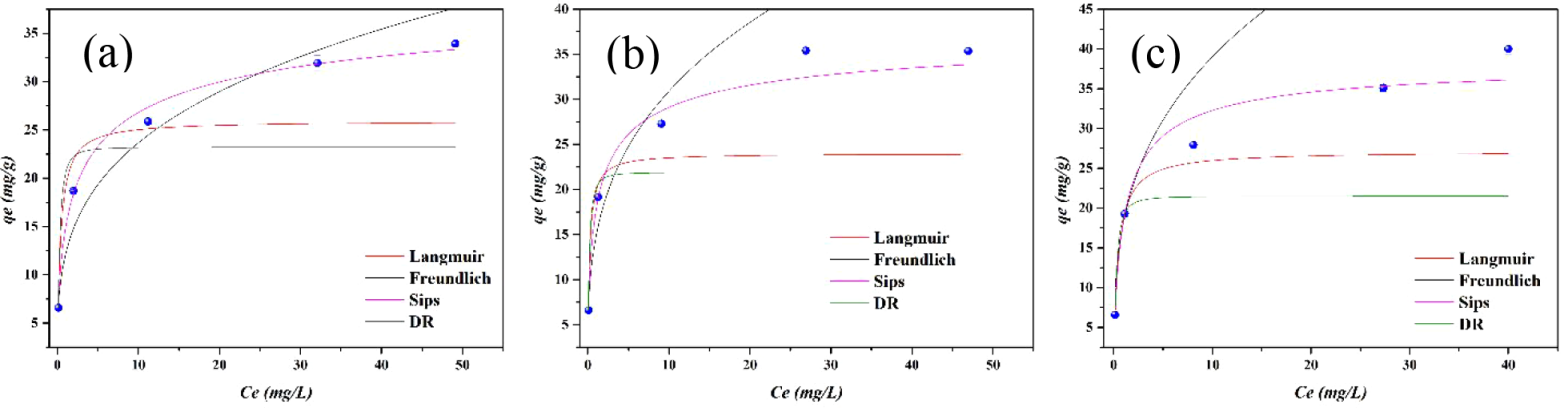
Adsorption isotherms for (a) 30 °C, (b) 40 °C and (c) 50 °C.

From Figure 15 and Table S5, it can be concluded that the experimental results fit best to the Sips adsorption isotherm model for the three temperatures studied, as this model exhibited the highest values of *R*_adj_^2^ and the lowest values of RMSE. Furthermore, this model provided the values closest to the theoretical maximum adsorption capacity (*q*_m_ = 41.00, 38.33, 38.64) compared to the experimental maximum adsorption capacity (*q*_m___exp_ = 33.93, 35.35, 40.00).

The values of *n* presented in the model indicate that the smaller and more distant they are from 1, the greater the heterogeneity of the surface. Thus, the adsorption of phosphate on the surface of CaFe can be characterized as a heterogeneous process, involving a distribution of adsorption energies across different active sites. This characteristic is also supported by the fact that the values of *q*_m_ in the Sips model are higher than those in the Langmuir model, suggesting the formation of multilayers. Such behavior reflects the coexistence of both homogeneous and heterogeneous adsorption domains, consistent with the structural complexity of the material.

The D-R model, although not as well fitted as the Sips model, still provides valuable insights, particularly regarding the mean adsorption energy. The values obtained, all exceeding 16 kJ/mol, strongly suggest the presence of stronger interactions between phosphate ions and the CaFe surface. These findings indicate that, in addition to physisorption, other mechanisms such as ion exchange and chemisorption may also play a role, highlighting the complexity of the adsorption process.

### 3.5 Adsorption Thermodynamics

Temperature variation influences the kinetic energy and mobility of the adsorbate species, primarily affecting the equilibrium constant. Therefore, temperature variation in a process can alter the adsorption capacity. For the thermodynamic study (Table S6, the equilibrium constant values from the Sips model (*k*_S_) were used, as this model provided the best fit to the experimental data.

From Table S6, it can be concluded that the adsorption of phosphate onto the CaFe surface is a spontaneous and favorable process, as evidenced by the negative values of Δ*G*°. The positive value of Δ*H*° confirms that the process is endothermic, indicating that higher temperatures enhance adsorption. In addition, the positive Δ*S*° values suggest an increase in system randomness during adsorption. This increase in entropy likely arises from the displacement of structured water molecules from the solvation layer of the phosphate ion and the adsorbent surface, as well as from the release of counterions previously associated with the surface.

The formation of inner-sphere complexes (direct P–O–Fe/Ca bonding) tends to promote the loss of the strongly ordered hydration surrounding the adsorbate and the surface, releasing water molecules and ions, which increases the number of microstates in the system and makes the −*T*Δ*S*° term sufficiently favorable to offset the positive Δ*H*°. Local reorganizations in the coordination network of the surface (creation of coordination sites, partial removal of coordinated water) may also contribute to the observed entropic gain.^50,51^

Moreover, the value of Δ*H*° < 20 kJ/mol indicates that the process occurs through physisorption, involving weak electrostatic interactions and hydrogen bonding rather than strong chemical bonding. These results are consistent with the *q*_m_exp_ values presented in Table S5, as the adsorption capacity increases with temperature. Similar results were observed by Brontowiyono et al. and Gu et al. when analyzing the removal of phosphate from aqueous solutions using manganese and zinc ferrites, respectively.^25,26^

### 3.6 Coexistence of Anions

The analysis of anion coexistence is an essential parameter for evaluating the selectivity of an adsorbent. This study allows for the examination of competition between anions for adsorption sites and helps identify which anions exert the greatest influence on the adsorption of a specific adsorbate. Figure 16 illustrates the effect of anion coexistence on the adsorption of phosphate on the CaFe surface.

**Figure 16:**
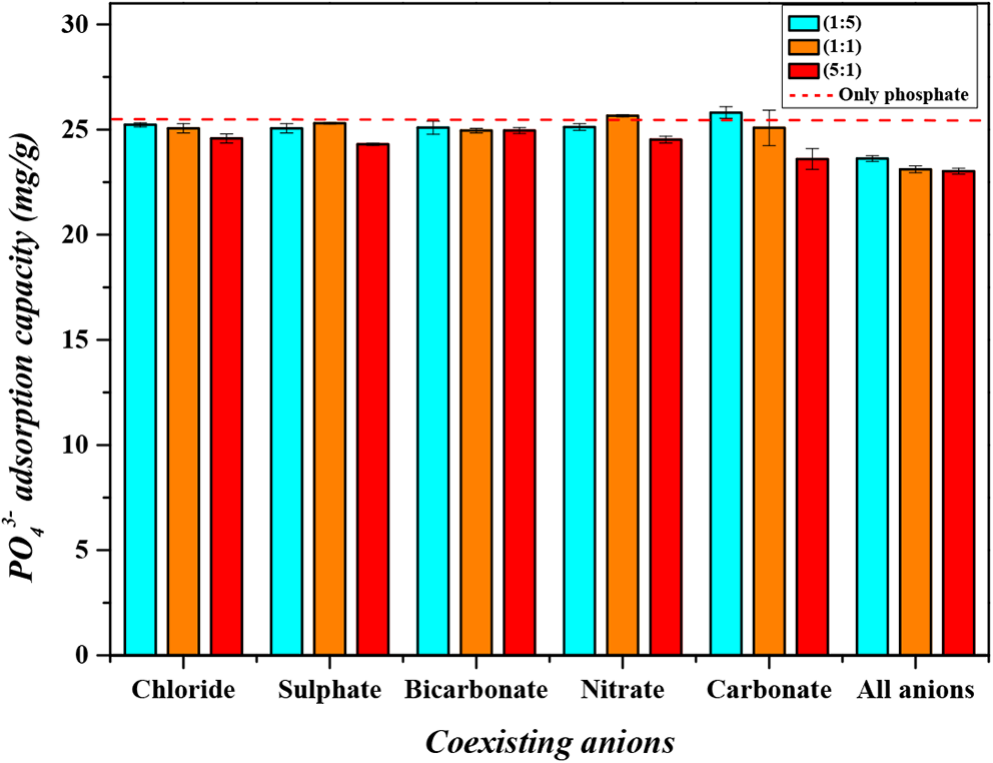
Coexistence of anions.

Based on Figure 16, it can be concluded that the adsorption of phosphate is minimally affected by the presence of the other anions analyzed, even with variations in the ratio of their concentrations. The blue bars represent solutions where the concentration of phosphate is five times higher than that of the other anions; the orange bars correspond to solutions where the concentrations of phosphate and the other anions are equal; and the red bars indicate solutions where the concentration of phosphate is five times lower. This behavior indicates a strong preference of CaFe for phosphate ions, which can be attributed to the high affinity of surface hydroxyl groups and Ca–Fe active sites for oxyanions with a higher charge density and stronger complexation ability, such as phosphate. Among the anions evaluated, carbonate had the greatest influence on the process. This effect can be explained by the fact that carbonate ions compete directly with phosphate for adsorption sites. At an initial pH of 4, phosphate is predominantly present as H_2_PO_4_^–^, whereas carbonate occurs mainly as H_2_CO_3_. However, the presence of carbonate gradually increases the solution pH to values between 8 and 8.5. Within this range, the predominant carbonate species becomes HCO_3_^–^. Consequently, a competition arises between H_2_PO_4_^–^ and HCO_3_^–^ for the active sites on CaFe, thereby reducing the phosphate adsorption capacity.

In addition to the study conducted with only two anions in solution, a scenario in which all the anions were present was also analyzed. Even under this condition, CaFe maintained its ability to adsorb phosphate, with reductions of less than 10%. This performance demonstrates the excellent selectivity and robustness of CaFe toward phosphate removal in complex water matrices. Such selectivity is particularly relevant for practical applications, where multiple anions coexist, as it ensures that the removal efficiency of phosphate is not significantly compromised.

### 3.7 Desorption Tests

The results in Table S7 demonstrate that the desorption efficiency of phosphate from CaFe strongly depends on the type and concentration of the eluent used. NaOH was the most effective desorption reagent, reaching efficiencies of 95.02% and 69.62% for concentrations of 0.5 and 0.1 mol/L, respectively. This high performance can be attributed to the ability of OH^–^ to compete strongly with phosphate ions for adsorption sites. The OH^–^ ions disrupt the electrostatic interactions and ligand exchange mechanisms responsible for phosphate binding to Ca–Fe surface sites, thereby promoting efficient desorption.

In contrast, NaCl solutions (0.5 and 0.1 mol/L) exhibited much lower desorption efficiencies (around 19%), which indicates that Cl^–^ ions have a weak affinity for the active sites of CaFe and are not capable of effectively displacing phosphate ions. Similarly, water alone showed the lowest desorption efficiency (14.70%), confirming that simple dilution or weak ionic exchange is insufficient to release phosphate ions strongly bound to the adsorbent surface. Gu and colleagues observed similar results for phosphate adsorption on zinc ferrite.^26^

Furthermore, it was observed that with each regeneration cycle (adsorption–desorption), there is a reduction in the amount of CaFe due to material loss during the transition between the stages. This loss primarily occurs during the separation and drying processes, which are necessary for the regeneration of the adsorbent. Although this factor is rarely mentioned in regeneration studies for batch processes, it is inevitable, making regeneration in such systems a greater challenge compared to processes in fixed-bed column systems, for example.

According to Figure 17, CaFe maintained an adsorption capacity above 24 mg/g for three cycles, exhibiting only a 7% decrease after this period. In the fourth cycle, the adsorption capacity decreased to 20 mg/g, corresponding to a 23% reduction relative to the initial value. Therefore, CaFe can be reused for up to three adsorption cycles without significant loss of efficiency, demonstrating substantial reusability. The possible causes for the reduction in adsorption capacity after the four cycles include the blockage of active sites, structural alterations in the CaFe particles, and irreversible adsorption through strong interactions or the formation of stable complexes.

**Figure 17:**
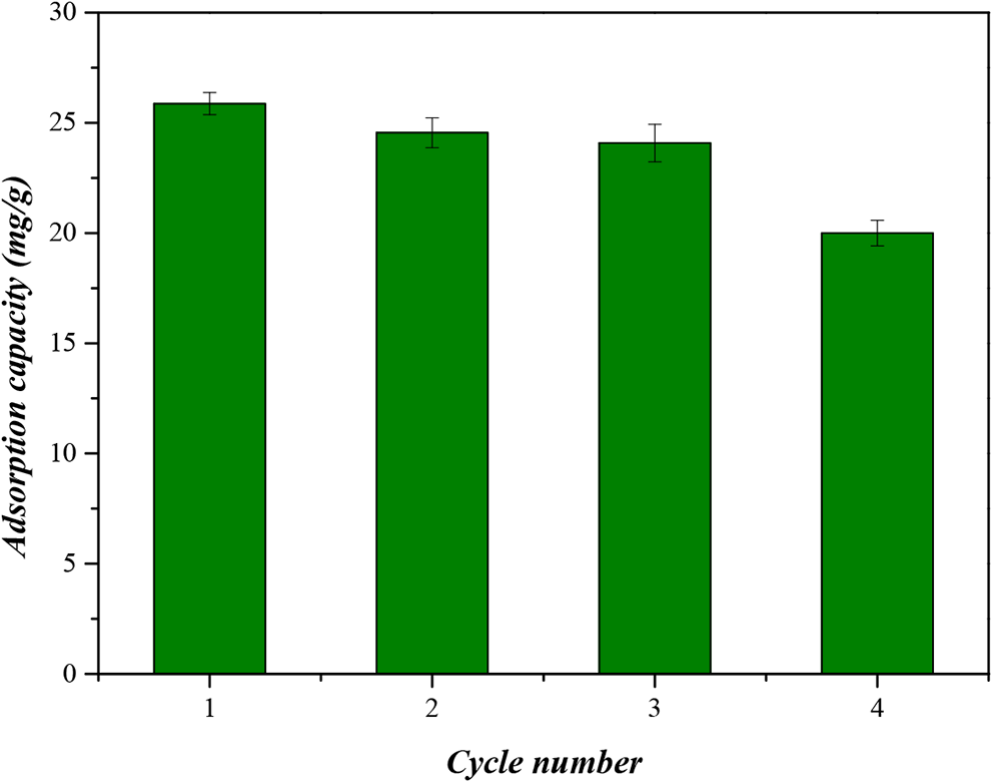
Regeneration of adsorption capacity.

### 3.8 Adsorption Mechanisms

The adsorption of phosphate onto the CaFe surface can be attributed to a combination of physisorption and chemisorption processes, with evidence suggesting that multiple mechanisms act simultaneously to govern the overall removal efficiency.

The thermodynamic parameters estimated using the Van’t Hoff equation indicated a relatively low enthalpy, suggesting a significant contribution from physisorption. However, the analysis of the *E* value provided by the DR model, along with the modifications observed in the FTIR spectrum, indicates the presence of higher-energy interactions. This apparent discrepancy can be explained by a mixed mechanism: the initial phosphate removal is governed by electrostatic attraction (physisorption, pH < pH_PZC_).

Physisorption primarily occurs through electrostatic interactions between negatively charged phosphate ions (H_2_PO_4_^–^) and positively charged sites on the CaFe surface, particularly at lower pH values where protonation of surface hydroxyl groups enhances electrostatic attraction. When the pH of the medium equals or exceeds the pH_PZC_ value, chemisorption mechanisms are initiated.

Chemisorption, on the other hand, plays a critical role in the stabilization and selectivity of phosphate adsorption. Inner-sphere complexation occurs when phosphate ions directly replace coordinated water molecules and bind with Ca^2+^ or Fe^3+^ active sites, leading to the formation of strong surface complexes. This mechanism is supported by the presence of characteristic phosphate-related bands in FTIR spectra, as well as by thermodynamic analyses indicating favorable adsorption. Ion exchange may also contribute, where H_2_PO_4_^–^/HPO_4_^2–^ replaces OH^–^ or HCO_3_^–^ groups associated with the CaFe surface. Additionally, surface precipitation cannot be ruled out, as phosphate may directly interact with calcium and iron active sites on the CaFe surface, leading to the formation of insoluble calcium–phosphate or iron–phosphate phases that enhance overall uptake.

The coexistence of these mechanisms—electrostatic attraction, inner-sphere complexation, ion exchange, and surface precipitation—highlights the complexity of phosphate adsorption on CaFe. Such multifaceted interactions have also been reported for other metal-modified adsorbents where chemisorption through inner-sphere association predominates but is often complemented by electrostatic contributions.^12,13,15,19,21–27,30–33,35,36,42,43,52,53^

Overall, despite the partial contribution of physisorption, the predominant mechanism governing phosphate removal by CaFe is chemisorption. This conclusion is consistent with previous studies on Fe/Ca-based adsorbents, which reported that phosphate uptake mainly occurs through chemical interactions such as inner-sphere complexation and surface precipitation, leading to the formation of stable Fe–P and Ca–P species.^51,53,54^

The strong evidence from DR modeling (*R* ^2^ > 0.86), FTIR analysis, and the observed persistence of adsorbed phosphate further corroborate that chemisorption dominates the overall process. Therefore, CaFe acts not only as a high-capacity adsorbent but also as a reactive substrate promoting the formation of stable phosphate complexes and precipitates, which account for its high selectivity and removal efficiency. Figure 18 illustrates the adsorption mechanisms.

**Figure 18:**
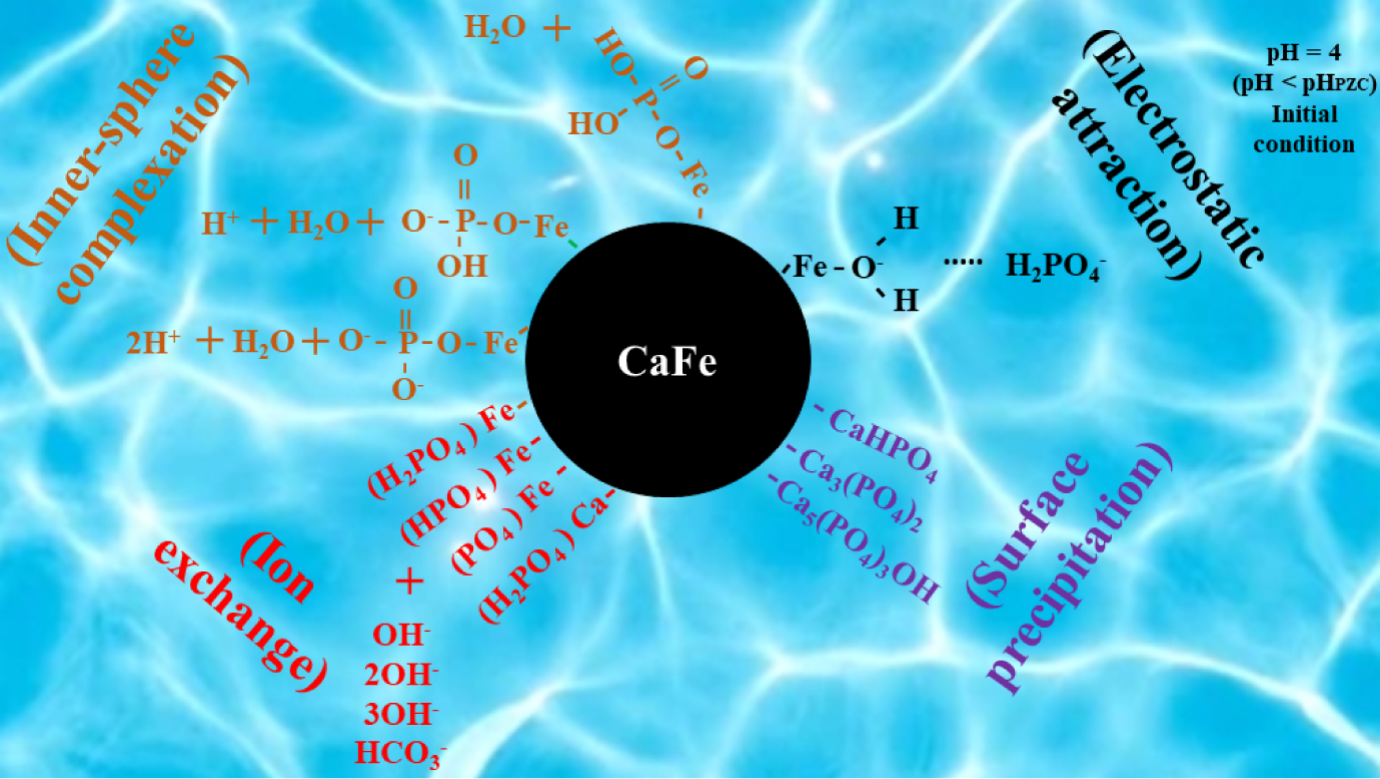
Adsorption mechanisms.

### 3.9 Application in Real Effluent

Table S8 demonstrates the applicability of CaFe in treating a real slaughterhouse effluent. The raw effluent presented slightly alkaline conditions (pH ≈ 8.3), high conductivity, elevated turbidity, and considerable phosphate concentration, which are typical characteristics of this type of wastewater.

Based on Table S8, the results clearly indicate that CaFe is not only effective in reducing phosphate levels but also contributes to the overall improvement of effluent quality by decreasing conductivity and turbidity. This behavior can be attributed to the simultaneous adsorption of other dissolved ions and suspended solids, which enhances the multifunctional role of CaFe as a treatment material.

A key factor influencing phosphate removal was the solution pH. Without pH adjustment, CaFe achieved a removal efficiency of 53.63%. However, when the effluent was acidified to pH 4 prior to adsorption, the efficiency increased markedly to 85.06%. This result highlights the importance of pH in modulating surface charge properties and adsorption mechanisms, particularly favoring electrostatic attraction and surface complexation under acidic conditions.

Under neutral or slightly basic conditions, the surface of CaFe is already slightly negative relative to its pH_PZC_, reducing electrostatic attraction and explaining the observed decrease in efficiency. Moreover, adsorption promoted by the calcium present in CaFe may occur through pH-dependent pathways. At neutral pH, the formation of calcium phosphate phases may take place depending on the availability of Ca^2+^ and the degree of saturation. However, when the Ca^2+^ activity is low or the saturation condition is not reached, the contribution from precipitation will be limited, and removal will depend primarily on adsorption at Fe–O/Fe–OH sites. Recent studies have reported similar results.^30,54–57^

These findings demonstrate that CaFe is a promising adsorbent for the remediation of real effluents with complex matrices, such as slaughterhouse wastewater. Furthermore, if stricter discharge limits are required, the removal performance could be further optimized by adjusting operational parameters such as adsorbent dosage, which would increase the number of available active sites for phosphate binding.

### 3.10 Comparison with Other Studies

Table S9 presents a comparison between the phosphate adsorption capacity of the CaFe material and the values reported in various studies from the literature.

Based on the Table S9, it is observed that CaFe exhibits a phosphate adsorption capacity comparable to that of other previously investigated materials, highlighting its potential as an adsorbent for practical applications. It is also noteworthy that CaFe offers significant advantages over several adsorbents described in the literature, particularly regarding its simple synthesis, which relies on low-cost precursors, wide availability, and reduced environmental impact. These characteristics contrast with more complex synthesis routes, which often require stringent experimental conditions, high-cost precursors, and pose a greater risk of environmental waste generation if improperly disposed of.

## 4 Conclusions

This study demonstrated that CaFe, synthesized via a simple coprecipitation route, is a highly effective and sustainable adsorbent for phosphate removal from aqueous media. The material exhibited remarkable performance, with a maximum experimental adsorption capacity (*q*_m_exp_ = 40 mg/g), high selectivity against competing anions, and excellent regeneration efficiency (95.02%) using 0.5 mol/L NaOH as the eluent.

Characterization analyses revealed that the superior adsorption performance is directly related to the physicochemical properties of CaFe, including its rough surface morphology, high density of active sites, thermal stability, and predominance of an amorphous phase. These features favor strong phosphate interactions and contribute to the robustness of the material. Furthermore, optimization through response surface methodology (RSM) confirmed the reliability of the adsorption process (*R*_adj_^2^ = 0.97), indicating that the ideal synthesis conditions are precipitation at 90 °C and calcination at 313 °C for 4 h.

Kinetic and equilibrium studies revealed that phosphate adsorption is best described by the PSO model and the Sips isotherm, evidencing multilayer adsorption in a heterogeneous system. Thermodynamic parameters further confirmed the spontaneous and endothermic nature of the process, suggesting the contribution of both physisorption and chemisorption mechanisms.

Importantly, the real effluent application demonstrated that CaFe is not only efficient in phosphate removal but also in reducing turbidity and conductivity, highlighting its multifunctionality in complex water matrices. The performance was significantly enhanced under acidic conditions (initial pH = 4), achieving removal efficiencies above 80%.

Overall, CaFe emerges as a promising and eco-friendly alternative for phosphate remediation. Unlike other adsorbents based on toxic metals such as Mn, Zn, Cu, La, and Ce, the CaFe system combines high efficiency, easy regeneration, and environmental safety, making it a viable material for practical wastewater treatment applications.

## Supplementary Material



## Notes

DOE for factor analysis (Table S1); ANOVA for factor analysis (Table S2); experimental and predicted values for CaFe adsorption capacity (Table S3); results of adsorption kinetic modeling (Table S4); results of modeling adsorption isotherms (Table S5); adsorption thermodynamic parameters (Table S6); eluent tests (Table S7); application of CaFe in slaughterhouse effluent (Table S8); comparison of CaFe with other adsorbents (Table S9) (PDF)
