# Supplementary material for: Eco-Friendly and Easily Synthesized Amorphous Fe–Ca (Oxy)hydroxide for Selective Phosphate Removal from Synthetic and Real Effluents: Synthesis, Optimization, and Application
Source: ACS Omega. 2026 Jan 26;11(5):7818–32. doi: 10.1021/acsomega.5c09613 (PMC12903001; doi:10.1021/acsomega.5c09613)
Supplement: Supplementary file 1 [file ao5c09613_si_001.pdf]

# Eco-friendly and easily synthesized amorphous Fe–Ca (oxy)hydroxide for selective phosphate removal from synthetic and real effluents: synthesis, optimization, and application

Yago Neco Teixeira[a*], Elias Matias Bentes[b], Jackson Evangelista[c], Daniel Bernardes Silva[c], Jorge Marcell Coelho Menezes[c], Thiago Mielle Brito Ferreira Oliveira[c], Raimundo Nonato Pereira Teixeira[a], Ronaldo Ferreira do Nascimento[d], Francisco José de Paula Filho[c]

[a]*Biological Chemistry Department, Regional University of Cariri, R. Cel. Antonio Luis, 1161, Crato, CE 63105-000, Brazil.*
[b]*Materials Engineering Section (SE/8), Military Institute of Engineering, R. Praça Gen. Tibúrcio, 80, Rio de Janeiro, RJ 22290-270, Brazil.*
[c]*Science and Technology Center, Federal University of Cariri, Av. Ten. Raimundo Rocha, 1639, Juazeiro do Norte, CE 63048-080, Brazil.*
[d]*Analytical and Physical Chemistry Department, Federal University of Ceará, Fortaleza, CE 60356-000, Brazil.*
[*]Email: yago.neco@urca.br

Table S1: DOE for factor analysis.

| Run Order | Real values | | | Response |
|---|---|---|---|---|
| | **A** | **B** | **C** | **Adsorption capacity (mg/g)** |
| 1 | 30 | 400 | 3 | 15.23 ± 0.84 |
| 2 | 60 | 300 | 3 | 19.39 ± 0.61 |
| 3 | 60 | 400 | 4 | 19.39 ± 0.74 |
| 4 | 60 | 300 | 3 | 19.14 ± 0.87 |
| 5 | 90 | 300 | 4 | 21.82 ± 0.03 |
| 6 | 60 | 200 | 2 | 20.74 ± 0.60 |
| 7 | 60 | 300 | 3 | 19.05 ± 0.50 |
| 8 | 60 | 400 | 2 | 15.11 ± 0.96 |
| 9 | 90 | 300 | 2 | 21.09 ± 0.18 |
| 10 | 30 | 200 | 3 | 19.49 ± 0.60 |
| 11 | 30 | 300 | 2 | 18.67 ± 0.27 |
| 12 | 90 | 200 | 3 | 20.24 ± 0.13 |
| 13 | 60 | 300 | 3 | 19.19 ± 0.73 |
| 14 | 60 | 200 | 4 | 20.04 ± 0.13 |
| 15 | 90 | 400 | 3 | 19.06 ± 0.35 |
| 16 | 30 | 300 | 4 | 18.90 ± 0.20 |
| 17 | 60 | 300 | 3 | 19.19 ± 0.19 |

*Adsorption parameters: CaFe = 1.5 g/L; $PO_4^{3-}$ = 50 mg/L; 150 rpm; 90 min; 20 °C.

Table S2: ANOVA of factor analysis.

| Source | DF | SS | MS | F-value | p-value |
|---|---|---|---|---|---|
| Model | 9 | 46.55 | 5.17 | 33.51 | 1.421E-12 |
| Linear | 3 | 32.05 | 10.68 | 69.20 | 1.440E-4 |
| A | 1 | 12.30 | 12.30 | 79.69 | 4.498E-5 |
| B | 1 | 17.17 | 17.17 | 111.23 | 1.505E-5 |
| C | 1 | 2.58 | 2.58 | 16.69 | 0.005 |
| Quadratic | 3 | 5.87 | 1.96 | 12.68 | 0.001 |
| $A^2$ | 1 | 0.40 | 0.40 | 2.56 | 0.153 |
| $B^2$ | 1 | 4.16 | 4.16 | 26.92 | 0.001 |
| $C^2$ | 1 | 1.63 | 1.63 | 10.54 | 0.014 |
| 2 Factors interaction | 3 | 8.63 | 2.88 | 18.64 | 0.003 |
| AB | 1 | 2.37 | 2.37 | 15.36 | 0.006 |
| AC | 1 | 0.06 | 0.06 | 0.40 | 0.545 |
| BC | 1 | 6.20 | 6.20 | 40.17 | 3.898E-4 |
| Residual | 7 | 1.08 | 0.15 | | |
| Lack of fit | 3 | 1.02 | 0.34 | 21.87 | 0.006 |
| Pure error | 4 | 0.06 | 0.02 | * | * |
| Total | 16 | 47.63 | | | |

$R^2 = 0.9773$. $R_{adj}^2 = 0.9481$. $R_{pred}^2 = 0.6559$.

Table S3: Experimental and predicted values for CaFe adsorption capacity.

| Run | Precipitation temperature (°C) | Calcination temperature (°C) | Calcination time (h) | Actual value (mg/g) | Predicted value (mg/g) |
|---|---|---|---|---|---|
| 1 | 30 | 400 | 3 | 15.23 | 14.83 |
| 2 | 60 | 300 | 3 | 19.39 | 19.30 |
| 3 | 60 | 400 | 4 | 19.39 | 19.29 |
| 4 | 60 | 300 | 3 | 19.14 | 19.30 |
| 5 | 90 | 300 | 4 | 21.82 | 21.75 |
| 6 | 60 | 200 | 2 | 20.74 | 21.11 |
| 7 | 60 | 300 | 3 | 19.05 | 19.30 |
| 8 | 60 | 400 | 2 | 15.11 | 15.66 |
| 9 | 90 | 300 | 2 | 21.09 | 20.61 |
| 10 | 30 | 200 | 3 | 19.49 | 19.33 |
| 11 | 30 | 300 | 2 | 18.67 | 18.12 |
| 12 | 90 | 200 | 3 | 20.24 | 20.28 |
| 13 | 60 | 300 | 3 | 19.19 | 19.30 |
| 14 | 60 | 200 | 4 | 20.04 | 19.77 |
| 15 | 90 | 400 | 3 | 19.06 | 18.85 |
| 16 | 30 | 300 | 4 | 18.9 | 19.27 |
| 17 | 60 | 300 | 3 | 19.19 | 19.30 |

Table S4: Results of adsorption kinetic modeling.

| Adsorbent | Dosage (g/L) | $PO_4^{3-}$ conc. (mg/L) | | PFO | | | | PSO | | | | Elovich | | | |
|---|---|---|---|---|---|---|---|---|---|---|---|---|---|---|---|
| | | | $q_{e\_exp}$ (mg/g) | $q_e$ (mg/g) | $k_1$ (1/min) | $R_{adj}^2$ | RMSE | $q_e$ (mg/g) | $k_2$ (g/mg.min) | $R_{adj}^2$ | RMSE | α (mg/g.min) | β (mg/g) | $R_{adj}^2$ | RMSE |
| CaFe | 1.5 | 10 | 6.63 | 6.47 ± 0.07 | 0.61 ± 0.06 | 0.9929 | 0.1563 | 6.63 ± 0.02 | 0.26 ± 0.01 | 0.9996 | 0.0333 | 2.29E8 ± 6.99E8 | 3.76 ± 0.51 | 0.9964 | 0.1105 |
| | 1.5 | 50 | 25.48 | 22.15 ± 1.06 | 0.21 ± 0.04 | 0.9087 | 2.0572 | 24.10 ± 0.81 | 0.01 ± 0.00 | 0.9696 | 1.1874 | 50. 95 ± 6.59 | 0.30 ± 0.01 | 0.9985 | 0.2603 |
| | 1.5 | 100 | 35.13 | 31.52 ± 1.65 | 0.23 ± 0.05 | 0.8873 | 3.239 | 34.15 ± 1.32 | 0.01 ± 0.00 | 0.9578 | 1.9827 | 105.04 ± 35.66 | 0.22 ± 0.01 | 0.9921 | 0.8583 |
| | 5 | 10 | 1.99 | 1.97 ± 0.00 | 1.53 ± 0.09 | 0.9999 | 0.0043 | 1.99 ± 0.00 | 13.59 ± 0.59 | 0.9999 | 0.0008 | 1.74E29 ± 3.77E30 | 37.32 ± 11.10 | 0.9982 | 0.0249 |
| | 5 | 50 | 9.91 | 9.71 ± 0.11 | 0.61 ± 0.06 | 0.9929 | 0.2380 | 9.95 ± 0.03 | 0.17 ± 0.01 | 0.9997 | 0.0471 | 3.94E8 ± 1.24E9 | 2.52 ± 0.35 | 0.9962 | 0.1732 |
| | 5 | 100 | 18.96 | 17.85 ± 0.45 | 0.38 ± 0.05 | 0.9672 | 0.9481 | 18.74 ± 0.24 | 0.04 ± 0.00 | 0.9938 | 0.4110 | 2941.41 ± 2057.93 | 0.63 ± 0.05 | 0.9954 | 0.3559 |

Table S5: Results of modeling of adsorption isotherms.

| Adsorbent | Temperature (°C) | | Langmuir | | | | | Freundlich | | | |
|---|---|---|---|---|---|---|---|---|---|---|---|
| | | $q_{m\_exp}$ (mg/g) | $q_m$ (mg/g) | $k_L$ (L/mg) | Radj² | RMSE | | $k_F$ (mg g-1 (mg L-1)-1/n) | n | Radj² | RMSE |
| CaFe | 30 | 33.93 | 25.94 ± 3.17 | 2.73 ± 0.46 | 0.9142 | 18.0248 | | 12.11 ± 0.51 | 3.43 ± 0.23 | 0.9397 | 15.1200 |
| | 40 | 35.35 | 24.01 ± 2.23 | 4.72 ± 0.63 | 0.9493 | 27.1757 | | 14.82 ± 0.96 | 3.13 ± 0.26 | 0.9239 | 33.2728 |
| | 50 | 40.00 | 27.15 ± 1.89 | 2.20 ± 0.51 | 0.9855 | 14.1274 | | 18.31 ± 0.72 | 3.04 ± 0.68 | 0.8650 | 43.1061 |
| | | | **Sips** | | | | | **Dubinin-Radushkevich** | | | |
| | | | $q_m$ (mg/g) | $k_S$ (L/mg) | N | Radj² | RMSE | $q_m$ (mg/g) | kDR (mol²/kJ²) | E (kJ/mol) | Radj² | RMSE |
| | 30 | 33.93 | 41.00 ± 3.29 | 0.57 ± 0.09 | 0.52 ± 0.03 | 0.9967 | 2.8639 | 23.22 ± 3.26 | 4.09E-8 ± 4.68E-9 | 3496.42 | 0.8642 | 22.6869 |
| | 40 | 35.35 | 38.33 ± 4.37 | 0.86 ± 0.21 | 0.56 ± 0.05 | 0.9950 | 6.9609 | 21.89 ± 2.16 | 2.62E-8 ± 2.24E-9 | 4368.52 | 0.9268 | 32.6497 |
| | 50 | 40.00 | 38.64 ± 3.17 | 0.91 ± 0.15 | 0.74 ± 0.04 | 0.9979 | 4.3724 | 21.48 ± 0.58 | 3.65E-8 ± 5.75E-9 | 3701.17 | 0.9675 | 21.1421 |

Table S6: Adsorption thermodynamics parameters.

| Fe-Ca composite | T (°C) | ΔG° (kJ mol$^{-1}$) | ΔH° (kJ mol$^{-1}$) | ΔS° (kJ mol$^{-1}$) | $R_{adj}^2$ | RMSE |
|---|---|---|---|---|---|---|
| CaFe | 30 | -55.38 | 17.38 ± 8.65 | 0.24 ± 0.025 | | |
| | 40 | -57.78 | | | 0.6564 | 3.34E8 |
| | 50 | -60.18 | | | | |

Table S7: Eluent tests.

| **Desorption Reagent** | **Concentration (mol/L)** | **Amount adsorbed (mg/g)** | **Amount desorbed (mg/g)** | **Desorption efficiency (%)** |
|---|---|---|---|---|
| NaOH | 0.5 | 22.28 ± 0.51 | 21.17 ± 0.73 | 95.02 |
| NaOH | 0.1 | 22.35 ± 0.46 | 15.56 ± 0.37 | 69.62 |
| NaCl | 0.5 | 22.90 ± 0.19 | 4.36 ± 0.24 | 19.04 |
| NaCl | 0.1 | 22.46 ± 0.43 | 4.36 ± 0.24 | 19.41 |
| $H_2O$ | - | 22.51 ± 0.16 | 3.31 ± 0.05 | 14.70 |

Table S8: Application of CaFe in slaughterhouse effluent.

| **Samples** | **Final pH** | **Conductivity (µS/cm)** | **Turbidity (NTU)** | **Temperature (°C)** | **Nitrate (mg/L)** | **Chloride (mg/g)** | **Sulphate (mg/g)** | **Bicarbonate (mg/g)** | **Phosphate (mg/L)** | **Phosphate removal (%)** |
|---|---|---|---|---|---|---|---|---|---|---|
| **Raw effluent** | 8.3 | 3020 ± 2.2 | 100 ± 0.8 | 23 | 0.7 ± 0.6 | 325 ± 15 | 254.2 ± 10 | 760 ± 15 | 35 ± 2.5 | - |
| **Treated effluent (no initial pH adjustment)** | 8.1 | 2285 ± 2 | 96 ± 1.2 | 23 | - | - | - | - | 16.23 ± 1.2 | 53.63 ± 2.5 |
| **Treated effluent (adjust initial pH = 4)** | 7.5 | 1841 ± 2 | 89 ± 2 | 23 | - | - | - | - | 5.23 ± 0.5 | 85.06 ± 1.5 |

*Adsorbent dosage: 1.5 g/L, 150 RPM.

Table S9: Comparison of CaFe with other adsorbents.

| Adsorbent | pH | Dosage (g/L) | Adsorption Capacity (mg/g) | References |
|---|---|---|---|---|
| **Metal-loaded orange waste** | 3 | 1.7 | 42.79 | [13] |
| **$MnFe_2O_4$** | 3 | 1.5 | 0.064 | [25] |
| **$ZnCe_{0.12}Fe_{1.88}O_4$** | 5.5 | 1 | 41.60 | [26] |
| **$MnFe_2O_4$** | 6 | 2.5 | 39.48 | [27] |
| **Ca-Mg modified iron oxide** | 7 | 12 | 17.00 | [33] |
| **LC-ZSM-5** | 5.5 | 2 | 45.00 | [52] |
| **LC-ZSM-5** | 5.5 | 2 | 47.10 | [53] |
| **CaFe** | 4 | 1.5 | 41.00 | This study |
